# Applying an intersectional lens to alcohol inequities: A conceptual framework

**DOI:** 10.1111/add.70130

**Published:** 2025-07-14

**Authors:** Sophie Bright, Charlotte Buckley, Daniel Holman, Hazel Squires, Naomi Greene, Nina Mulia, Carolin Kilian, Charlotte Probst, Colin Angus, John Holmes, Helena M. Constante, Meesha Warmington, Robin Purshouse

**Affiliations:** ^1^ Sheffield Addictions Research Group (SARG), Sheffield Centre for Health and Related Research (ScHARR), Faculty of Medicine, Dentistry & Health University of Sheffield Sheffield UK; ^2^ Department of Psychology, Institute of Population Health University of Liverpool UK; ^3^ Department of Automatic Control and Systems Engineering University of Sheffield UK; ^4^ Department of Sociological Studies University of Sheffield Sheffield UK; ^5^ Sheffield Centre for Health and Related Research (ScHARR), Faculty of Medicine, Dentistry & Health University of Sheffield Sheffield UK; ^6^ National Opinion Research Center (NORC) University of Chicago Chicago IL USA; ^7^ Alcohol Research Group Public Health Institute Emeryville CA USA; ^8^ Center for Interdisciplinary Addiction Research (ZIS), Department of Psychiatry and Psychotherapy University Medical Center Hamburg‐Eppendorf (UKE) Hamburg Germany; ^9^ Institute for Mental Health Policy Research Centre for Addiction and Mental Health Toronto Ontario Canada; ^10^ The School of Education University of Sheffield Sheffield UK

**Keywords:** alcohol, conceptual framework, inequities, intersectionality, substance use, theory

## Abstract

**Background:**

Prior research has demonstrated substantial inequities in alcohol consumption, alcohol‐related harms, and mortality. These inequities arise from a complex interplay of factors, unlikely addressed by single factor analyses or solutions. Conceptual frameworks, such as the National Institute on Minority Health and Health Disparities (NIMHD) Research Framework, aim to reflect this complexity and support multifaceted research and action. This paper adapts the NIMHD Framework to focus on alcohol‐related inequities and integrate core intersectionality principles.

**Method:**

We developed the Intersectional Alcohol Inequities Framework (IAIF) through collaboration among leading scholars in alcohol, intersectionality, and policy modelling. In a workshop centred on the core ideas of intersectional frameworks, we identified key factors influencing alcohol consumption and related harms, using the United States as a case study. Using thematic analysis, we grouped the discussion points, then mapped them against the NIMHD Framework. We searched the literature to expand upon workshop insights, iteratively refining the framework until reaching idea saturation.

**Results:**

To align with the core ideas of intersectionality, the IAIF introduced new elements absent in the NIMHD Framework, specifically a ‘power’ domain, a ‘historical’ level, and emphasis on relationality. We also incorporated a ‘digital environment’ domain, to reflect an important element of contemporary social context, as previously identified by other health equity scholars. We provided examples of their relevance to alcohol inequities, highlighted practical applications for stakeholders, and discussed adaptability to other public health issues and contexts.

**Conclusions:**

The Intersectional Alcohol Inequities Framework offers a tool for critical dialogue on how various factors, across multiple levels and domains, intersect to influence alcohol‐related outcomes. It can provide support and guidance for researchers, facilitate the identification of research needs and gaps in current policies, support the design of new policies and interventions, and inform comprehensive patient management.

## INTRODUCTION

### Alcohol inequities

Over 200 diseases, injuries and other health conditions have been causally linked to alcohol [1]. As of 2019, alcohol consumption was responsible for approximately 2.6 million deaths globally, and an estimated 7% of the world’s adult population lived with alcohol use disorders [2]. Prior research has demonstrated substantial inequities in alcohol consumption, alcohol‐related harms and mortality – with inequities defined as unfair differences in health between population groups, arising from the social conditions in which people are born, grow, live, work and age [3]. Alcohol inequities in consumption and harm have been observed in relation to gender, race, ethnicity, age, sexual orientation and socio‐economic status (SES), with minoritized populations — groups that are socially, politically or economically oppressed — often disproportionately affected [4, 5, 6, 7, 8, 9, 10, 11].

### An intersectional perspective

Alcohol inequities are shaped by a complex causal system involving interconnected factors at many levels. Intersectionality is a critical theoretical framework that embraces this complexity. Arising from Black feminist scholarship [12, 13, 14, 15], the term was originally coined by American civil rights and critical legal race scholar Kimberlé Crenshaw in 1989 [14]. Today, intersectionality is increasingly recognized as a vital concept in public health, with the potential to advance health equity [16, 17, 18].

According to Collins & Bilge [19] there are six core ideas of intersectional frameworks:

1. *Intersecting power relations* refers to the fact that power is relational, not static, and that power relations should be analysed both via their intersections (for example, of racism and sexism), and across domains of power.
2. *Social context* highlights how categories such as race and gender are socially constructed, with their relevance and influence changing over time and in different contexts.
3. *Relationality* emphasizes that socio‐demographic characteristics tied to social position/power, such as race, ethnicity, gender, sexuality and SES, do not exist in isolation but overlap and interact within systems of oppression, shaping distinct pathways through which individuals navigate and experience the world.
4. *Complexity* reflects the intersectionality goal of understanding complexity in the world, which requires complex analytic strategies and thinking.
5. S*ocial inequity* is emphasized as a multifaceted issue that cannot be explained by a single factor, but rather emerges from the interactions between various categories of power.
6. Finally, *social justice* underscores that intersectionality work should aim to dismantle inequity, not just document it.
7. Intersectionality can offer valuable insights into the complexity of alcohol inequities, and the results section shows how specific tenets have informed the Intersectional Alcohol Inequities Framework (IAIF) and/or their influence on alcohol inequities.

### The role of conceptual frameworks

**FIGURE 1:**
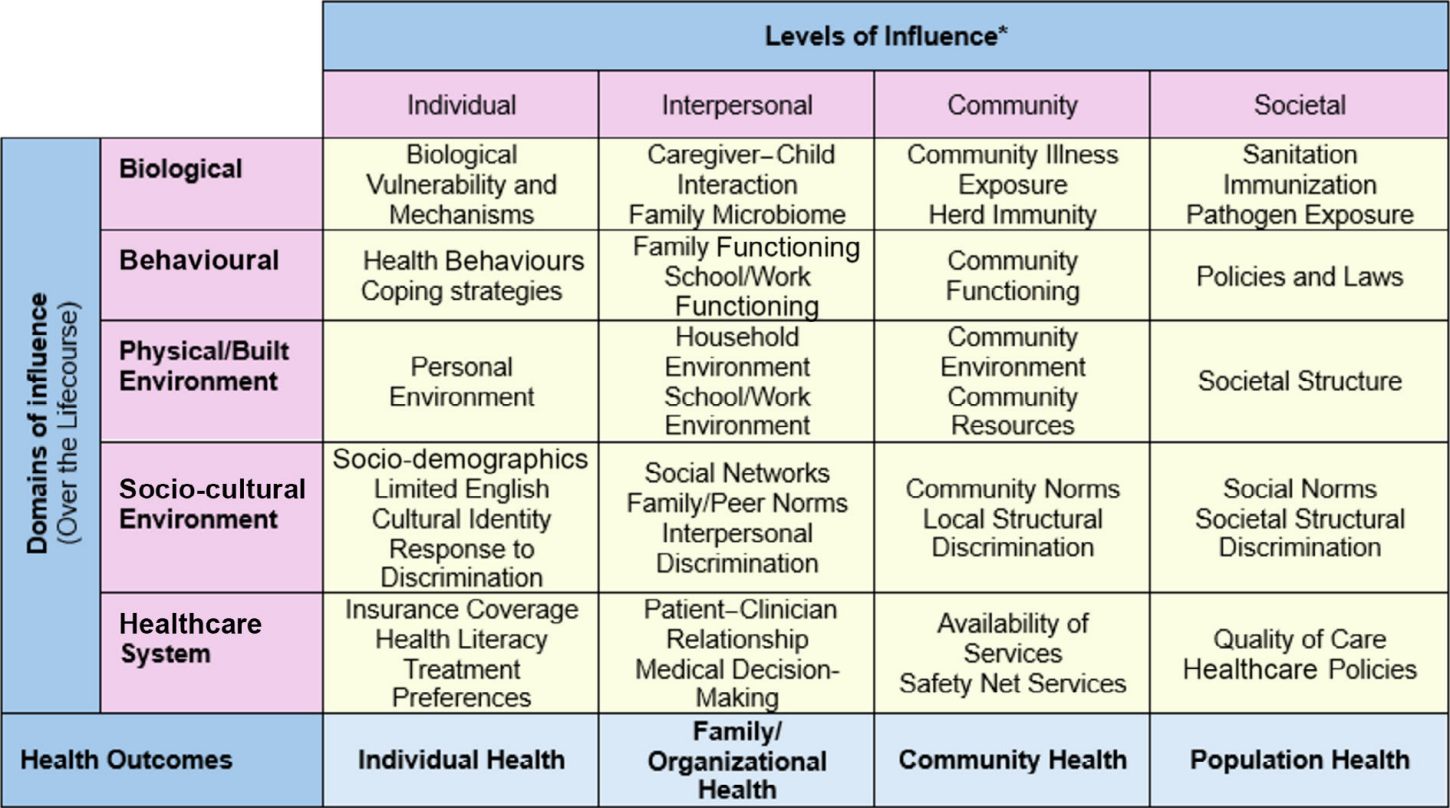
Reproduction of the National Institute on Minority Health and Health Disparities research framework [21]. *Health disparity populations: racial and ethnic minority groups, defined by Office of Management and Budget (OMB) directive 15; people with lower socio‐economic status; underserved rural communities; sexual and gender minority groups; and people with disabilities. Other fundamental chracateristics: sex and gender, disability and geopgraphic region.

Conceptual frameworks can be used to guide public health research, practice and policy. They are intentionally comprehensive, aiming to reflect the full ‘universe’ of factors relevant to a given issue [20]. One framework commonly used in Public Health is the National Institute on Minority Health and Health Disparities (NIMHD) research framework — a multi‐dimensional framework that highlights the wide range of health determinants relevant to understanding and addressing health disparities/inequities [21]. Drawing from previous frameworks [22, 23], it includes five domains of influence (biological; behavioural; physical environment; socio‐cultural environment; healthcare system) and four levels of influence (individual; interpersonal; community; societal), with examples of factors provided within each cell (Figure 1). The NIMHD framework has been adapted for some health problems (e.g. mental health disparities and vaccine hesitancy) and has been tailored to some groups (e.g. American Indians and Alaska Natives) [24]. However, no known adaptation exists in relation to alcohol‐related problems. While there are other frameworks summarizing the determinants of alcohol consumption and/or alcohol‐related harm (e.g. 29–31), no known framework has been found to comprehensively map the factors with an intersectional perspective.

### Aims

We aim to provide a conceptual framework that visually illustrates the many pertinent factors influencing alcohol consumption, alcohol‐related harm and alcohol inequities, spanning multiple levels and domains. It can aid efforts to reduce alcohol inequities by promoting relational, multi‐level thinking, and by highlighting understudied areas. We provide examples of how different groups may apply the IAIF to their work. Potential users include healthcare providers, community‐based organizations, researchers, public health modellers, policymakers and research funders.

Recognizing the importance of context specificity, this first version of the conceptual framework focuses on the USA as a case study, given the rising alcohol‐related mortality over the past 20 years in the USA, alongside persistent – and in some cases, growing – alcohol‐related inequities [25, 26, 27]. Additionally, many foundational ideas of intersectionality were catalysed by social movement activism in the USA. However, the broad components of the framework allow for systematic adaptation to different settings, as discussed later.

## METHODS: DEVELOPMENT OF THE FRAMEWORK

The IAIF was developed through an iterative process of collaboration with experts and supplementary literature searching. A 2‐hour online workshop with international research experts was held on 13 March 2023, to brainstorm factors influencing alcohol consumption and related health harms in the USA. Participants were purposefully selected based on their research expertise in intersectionality and/or alcohol inequities. They participated on a voluntary basis and were not compensated. While we recognize the value of involving a broader range of users (e.g. community organizations, policymakers and individuals with lived experience), the workshop included researchers at this stage, with the expectation that a broader range of users will test, refine and adapt the IAIF.

Researchers were identified through the authors’ networks and relevant publications (e.g. intersectional analyses of alcohol outcomes). All participants provided written informed consent, and ethical approval was granted by The University of Sheffield School of Health and Related Research ethics committee (ref. 050600). Of the 30 researchers invited, 12 participated (40%), all of whom are authors of this article. The workshop included small group discussions addressing: (i) why some groups drink more/less than others; (ii) why health harms vary at similar consumption levels; and (iii) how interlocking power relations influence these factors, with a focus on the US context. Participants were encouraged to reflect on the core ideas of intersectionality and consider how their own social positions might influence their perspectives before discussions. We used an interactive whiteboard to enable participants to contribute both verbally and in writing, and video‐recorded the discussions for later analysis. The first author (S.B.) grouped discussion points into themes and drafted a preliminary version of the IAIF, in collaboration with the steering group (S.B., R.P., C.B., D.H. and H.S.). Initially, we used an inductive approach, but as the topics aligned with the NIMHD framework [28], we opted to adapt this framework.

**FIGURE 2:**
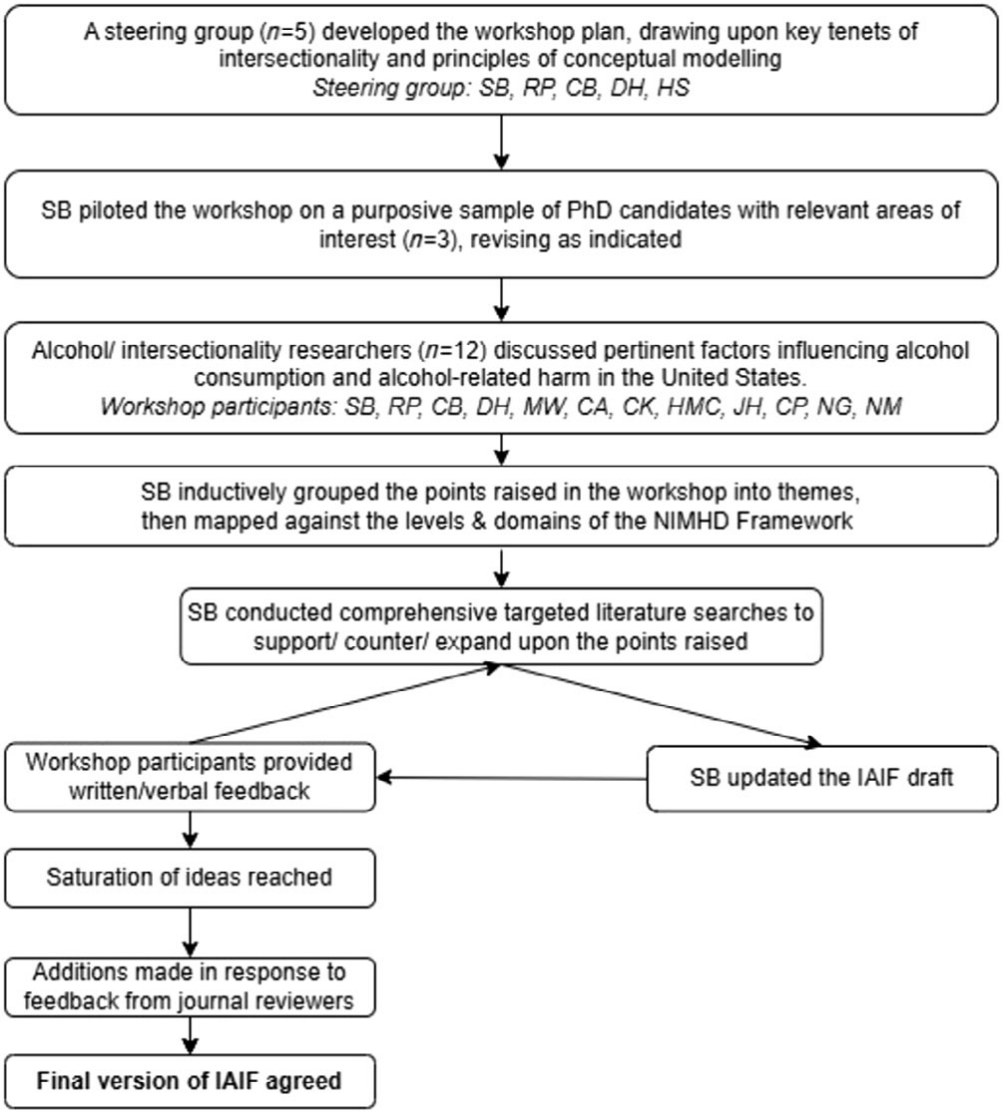
Overview of the Intersectional Alcohol Inequities Framework (IAIF) development process.

The IAIF was then refined, and additional factors identified, by iteratively consulting the literature and gathering feedback from the wider group. For example, one participant raised the lasting effects of historical redlining on healthcare service distribution, prompting further exploration into the impact of redlining on current alcohol outlet density [29]. Some levels within each domain were not covered in the initial workshop (e.g. ‘societal biological’ and ‘community behavioural’), prompting further consideration of these intersections during feedback rounds. We continued this process until we reached idea saturation, meaning that existing factors began to repeat, and no new factors were identified. Group feedback was gathered both via email and during a second online workshop, held on 22 April 2024. All participants provided written feedback, and nine participants (75%) also attended the second workshop. This process revealed gaps in the NIMHD framework, leading to the addition of a ‘power’ domain and a ‘historical’ level, plus a ‘digital’ domain, as previously suggested [30]. Some final additions were made to the IAIF in response to feedback during the journal review process. A diagrammatic overview of the IAIF development process is shown in Figure 2.

## RESULTS: THE INTERSECTIONAL ALCOHOL INEQUITIES FRAMEWORK

In this section we present the IAIF. We begin by outlining the broad structure of the IAIF, followed by an exploration of how the key tenets of intersectionality – power, social context (including the historical and digital environment), relationality, complexity, social inequity and social justice – are embedded in the framework and how they connect to alcohol‐related inequities.

**FIGURE 3:**
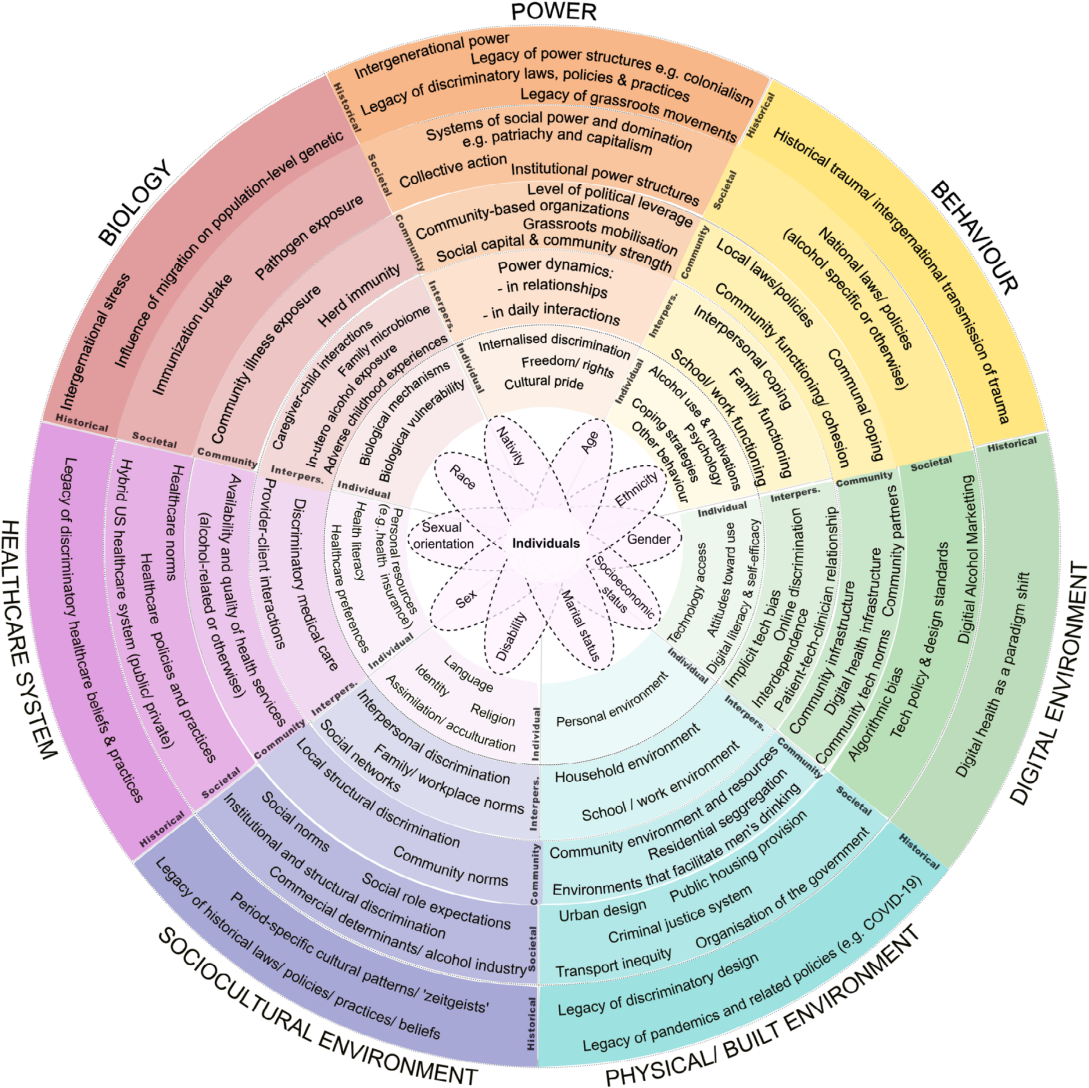
The Intersectional Alcohol Inequities Framework (IAIF). Please note, the social categories listed reflect some of those known to be associated with differences in alcohol consumption and related harm, but do not represent an exhaustive list.

The IAIF is presented in Figure 3. Individuals are at the centre, holding a unique social position of power/subordination and privilege/oppression shaped by overlapping social categories like race, gender, sexual orientation and SES. The social categories named in the diagram are examples, selected for their established associations with variations in alcohol consumption and related harm; however, they do not constitute an exhaustive list.

Surrounding the individual are five ‘levels of influence’ (individual, interpersonal, community, societal and historical) within seven ‘domains of influence’ (power, behaviour, digital environment, physical/built environment, socio‐cultural environment, healthcare system and biology). Key ‘factors’ contributing to alcohol consumption and/or harm are listed at the intersection of each level and domain. The dashed lines between levels and domains reflect that these factors do not exist in isolation, but rather they interact. Further details relating to each factor (e.g. descriptions, examples and references), are provided in the tables provided in Appendix S1.

### Power

Power is fundamental to intersectionality and influences alcohol consumption and related harm in multiple ways. Although power spans various levels and domains, we have positioned it as a distinct domain in the IAIF to highlight its role. Further, although alternative framings of power exist, for example, see work published by Collins in 2000 [31] and 2019 [32], we have used consistent levels across all domains for clarity, while integrating insights from other frameworks.

At the individual level, factors associated with power include personal freedoms, internalized discrimination and cultural pride. Personal freedoms encompass financial independence, rights (e.g. legal drinking age) and freedom from stigma/punitive controls [33].

Internalized discrimination refers to accepting beliefs, values and stereotypes about one’s group or about oneself, owing to group membership [34]. It may affect individuals belonging to any stigmatized group and may increase alcohol‐related harm by contributing to mental health conditions, such as depression [34, 35, 36, 37], which often co‐occur with harmful alcohol use [38, 39], as well as by creating barriers to seeking healthcare [40]. For instance, there is an established link between minority stressors (including internalized stigma) and alcohol use among sexual and gender minorities, particularly for certain subgroups [41]. Further, these groups experience higher rates of alcohol consumption and alcohol‐related problems compared with heterosexual cisgender individuals [41, 42]. In addition, alcohol consumption itself, and particularly alcohol use disorders (AUDs), may also contribute to internalized discrimination, more so for some groups, which may in turn affect alcohol treatment utilization [37, 43, 44].

An individual’s sense of cultural, racial or ethnic pride can also influence alcohol consumption. A strong sense of pride may protect against discrimination and mood disorders, though its effects vary across social categories. For example, among Latino adolescents, increased ethnic pride has been shown to directly reduce alcohol consumption in girls but not boys [45]. Further, for Asian and Black Americans, moderate identification with their racial/ethnic group has been shown to buffer the impact of discrimination on psychiatric disorders (including AUD), whereas for several other groups, high racial/ethnic identification intensifies this impact [46].

At the interpersonal level, power dynamics shape all relationships. For example, in healthcare, clinical interactions are inherently asymmetrical, with providers holding professional power and patients relying on them for care [47]. Intersecting socio‐demographic factors linked to social positions (e.g. gender or race), further influence these power imbalances. For example, Black American women have reported unequal treatment when seeking substance use treatment and feeling ‘silenced’ by healthcare providers [40]. Additionally, factors like healthcare literacy (healthcare domain, individual level), insurance coverage (healthcare domain, individual level) and comorbidities (biological domain, individual level) can further affect a patient’s negotiating power, reflecting the relational nature of the IAIF.

Power dynamics within intimate relationships are also important, particularly in heterosexual relationships where traditional gender roles and structural inequalities position men as dominant [48]. Harms from alcohol extend beyond the drinker, and there are clear gendered inequalities in who is affected by others’ drinking. Although men consume more alcohol and engage in riskier drinking patterns, women often disproportionately experience harms, ranging from intimate partner violence (IPV) to negative impacts on family functioning, mental health, social isolation and economic well‐being [49]. Male alcohol use is an established risk factor for IPV against women [50], while women’s heavier drinking can increase their risk of victimization [51]. Men’s drinking further affects children, contributing to family violence and emotional neglect [52]. In the USA, gender differences in alcohol's harm to others (AHTO) vary by the type of harm and the affected person’s own drinking [53, 54]. While other socio‐demographic factors – such as age and ethnicity – also influence these second‐hand effects [53], intersectional inequities in AHTO remains understudied.

At the community level, factors like uneven political leverage can undermine community power, while social capital, community‐based organizations and grassroots interventions can strengthen it. Affluent communities often have more political leverage owing to social status and influential connections [55], enabling them to attract resources and drive change, such as opposing unwanted developments like alcohol outlets or late‐night bars. However, disadvantaged communities can also build power to combat alcohol‐related harms. The ‘minority strengths model’ suggests that social support and community consciousness within minoritized populations can increase identity pride, resilience and self‐esteem, promoting healthy behaviours such as reduced alcohol use [56]. A systematic review further found that characteristics of social capital (community attachment, support and participation) are protective against alcohol use [57]. Moreover, minoritized communities, through community action, can challenge unjust social relations and alcohol‐related inequities. For example, grassroots movements have successfully changed alcohol policies in inner‐city neighbourhoods by passing ordinances regulating alcohol stores and protesting against targeted marketing from the alcohol industry. Additionally, collaborating with community organizations and applying participatory research approaches can support effective AUD prevention/treatment for marginalized populations by tailoring interventions to their specific needs and redistributing power back to community members [58, 59, 60].

At the societal level, factors include institutional power structures and collective action/social movements, which are embedded within broader intersecting systems of social power and domination, such as capitalism, white supremacy and patriarchy. For example, from a structural perspective, capitalism can drive harmful alcohol consumption through the aggressive marketing of alcohol, particularly in marginalized communities [61, 62]. Similarly, patriarchal norms shape gendered expectations, with men often socially rewarded for heavy drinking while women are more likely to be stigmatized for similar behaviour. Marriage, through its social and psychological impacts, appears to be generally protective against excessive alcohol use, especially for those married at younger ages [63]. However, as a gendered social institution, marriage can also reinforce power imbalances within heterosexual relationships, where men’s alcohol use poses a significant risk for IPV. Meta‐analyses have found that the association between alcohol and IPV victimization does not differ significantly by relationship status – including married, cohabiting, dating or divorced couples [50] – suggesting that the institution of marriage does not shield against alcohol‐related harm within intimate relationships. Further, the prohibition of same‐sex marriage is associated with higher rates of AUDs at the state level among lesbian, gay and bisexual populations, reflecting the mental health toll of this form of sexual orientation discrimination [64].

In relation to collective action, one recent notable social movement is the case of Black Lives Matter (BLM) [65]. Highly publicized anti‐Black violence has been associated with increased stress, anxiety and depression among Black Americans [66, 67]; however, engagement with BLM (via protest, social media, etc.) appears to be associated with positive emotions (such as hope and inspiration) and improved mental health, which in turn may influence alcohol consumption [68, 69, 70]. On the other hand, a systematic review of global studies on collective actions – such as protests, riots and revolutions – found evidence that such events, even when non‐violent, are associated with adverse mental health outcomes, including post‐traumatic stress disorder and major depression, again likely influencing alcohol consumption [71]. In the long term, increasing political pressure to tackle racism may reduce inequities in alcohol‐related harm through, for example, improved healthcare access and reductions in discriminatory policing.

Finally, factors at the ‘historical level’ (a newly introduced level, discussed below) include intergenerational power and the legacies of institutional power structures, such as colonialism, discriminatory laws and policies, and grassroots movements. For example, currently, alcohol outlet density, which has been positively associated with alcohol consumption and related harms [72, 73], is higher in poorer or more Black‐ and Hispanic‐populated neighbourhoods, despite a similar or lower demand for alcohol among these groups [74, 75]. This inequity in alcohol outlet density has arisen at least in part through historically racist urban land use practices, such as redlining, which systematically enforced racial segregation and disinvestment within certain communities. Recent studies utilizing geospatial data have shown that, despite the explicit outlawing of redlining in 1968 via the Fair Housing Act, alcohol outlets continue to be more densely distributed in historically redlined communities [29, 75]. Such practices reflect deeper colonial legacies, including the appropriation and control of land to marginalize racialized groups.

### Social context

When employing intersectionality as an analytical tool, it is important to contextualize inquiries and praxis, considering place and space (both geographic and digital), and the historical context [19]. Attending to context helps prevent the reification of social categories and inequities, while highlighting their links to broader intersecting systems of oppression and privilege, including capitalism, patriarchy and colonialism [76]. In this section, we draw attention to the influence of the historical context and the digital environment using the geographical context of the USA as a case study, for the aforementioned reasons.

#### Historical context

Social categories, their significance and their interaction with factors beyond the individual level, evolve over time. For example, the definition of gender, the number of recognized gender categories, societal gender norms and laws/policies related to gender have all evolved significantly in recent years across many countries. To draw explicit attention to the influence of such historical factors on alcohol inequities, we incorporated a ‘historical’ level into the IAIF.

For instance, in the behaviour domain we highlight the impact of historical trauma, defined as ‘cumulative emotional and psychological wounding over the lifespan and across generations, emanating from massive group trauma experiences’ [77]. In the US context, this is well documented within Native American communities in response to the brutal practices of colonization. Historical trauma can adversely affect the physical and mental health of descendants from historically oppressed groups and may lead to self‐destructive behaviours, including harmful alcohol use [77, 78]. While much of the US literature on historical trauma has focused on Native American populations, its effects can also be seen among other oppressed groups. For example, sexual minoritized groups may experience similar trauma responses owing to historical discrimination, including the criminalization and pathologization of homosexuality [79].

The influence of historical practices is also apparent in the healthcare domain. During colonial times, the justification for slavery and inhumane medical experimentation on Black individuals stemmed from the stereotype that they possess higher pain thresholds [80]. This belief persists today [81], and may contribute to Black people receiving inadequate treatment for pain [81] and mental health issues [82], both of which may motivate alcohol consumption [39, 83]. Furthermore, medical textbooks have historically over‐represented light skin tones [84], potentially leading to the misdiagnosis of conditions related to skin colour, such as jaundice (yellowing of the skin) in the context of liver cirrhosis.

#### Digital environment

Since the mid‐20th century, a global digital revolution has transformed society, bringing both benefits and risks to health and health inequities [85]. Recognizing this, it has been proposed that a digital environment domain be added to the NIMHD framework [30], which we also support, recognizing the role of the digital space in shaping alcohol‐related inequities.

A key example is the digitalization of health services. Digital alcohol interventions have shown some promise in relation to reducing harmful alcohol consumption [86], but usage of electronic health (eHealth) services varies across demographic groups [87, 88], owing to factors such as digital literacy (individual level), local digital infrastructure (community level), and tech policy and design standards, which influence content accessibility (societal level).

Another pertinent example is the growing influence of the alcohol industry in the digital space. Unlike non‐industry‐funded eHealth apps, industry‐funded apps may spread misinformation and subtly ‘nudge’ users toward continued alcohol use [89]. Further, alcohol marketing is shifting from traditional media to digital platforms, utilizing techniques like data‐driven targeting, user‐generated content, influencers, and combined advertising and purchasing strategies to more effectively promote and normalize drinking [90]. Exposure to online alcohol advertising is associated with higher alcohol consumption [91], yet this marketing is difficult to regulate because of its often discreet nature [90]. Of particular concern is exposure amongst young people [92], especially among Hispanic and Black youth, who seem to be disproportionately targeted [93, 94].

### Relationality

Fundamental to intersectionality is the understanding that social positions related to socio‐demographic categories (such as race, gender and sexual orientation) and systems of power overlap and interact, and should not be treated or studied separately [19]. This relationality is visually reflected in the IAIF by the overlapping of social categories at the centre of the framework, and by the dashed lines between all individual boxes.

It has long been recognized that socio‐demographic categories are linked to levels of alcohol consumption and associated harms. For instance, studies indicate that men tend to drink more than women [9] and that younger individuals generally consume more alcohol than older adults [95]. Drinking prevalence is consistently higher amongst those with high SES [11, 96], whilst alcohol‐attributable mortality is higher in those with low SES, partly explained by differences in drinking patterns [97]. Among racial and ethnic groups, non‐Hispanic white individuals are the most likely to be current drinkers, while non‐Hispanic American Indian/Alaska Native people have the highest rates of binge drinking and heavy drinking [98]. Additionally, those with low SES and individuals from Native American, Black and Hispanic communities experience disproportionately greater harm [99].

While these trends provide valuable insights into alcohol consumption patterns, the realities are much more complex. Social categories and systems of oppression interact in complex ways that cannot be captured through a single‐axis perspective [17]. Quantitative intercategorical studies can shed light on the complexities of how these factors interact [100, 101, 102, 103]. For example, Bright *et al*. [100] used an intersectional multilevel analysis of individual heterogeneity and discriminatory accuracy (MAIHDA) approach to quantify inter‐categorical disparities in alcohol consumption in the US along dimensions of sex, race and ethnicity, age, and education. They found that those holding multiple privileged positions tended to drink more, while those holding multiple marginalized positions drank less; young, highly educated white men were the most likely to be current drinkers and drank the most, whilst racially and ethnically minoritized women with lower education were the least likely to drink and drank the least, across all age categories. However, for groups with a mix of privileged and marginalized positions, alcohol consumption did not follow a simple or consistent pattern. Further, there were significant interaction effects between socio‐demographic dimensions for many intersectional strata, with several understudied groups found to have differing consumption to what would be expected based on additive effects alone. For example, failing to account for interaction effects in relation to alcohol consumption would significantly overestimate the consumption of young (21–24 years) Black and white men with low education, but underestimate the average consumption of adult (25–29 years) Black and Hispanic men with low education.

It is important to consider how factors across various domains and levels impact individuals within different social categories. This can be supported by integrating the IAIF with other relevant frameworks, such as the Capability, Opportunity and Motivation model for understanding behaviour (COM‐B) [104]. For instance, consider inequities in the utilization of alcohol‐related healthcare. In the USA, Latina/Hispanic women face particularly low odds of receiving alcohol screening and brief intervention [102] or utilizing alcohol treatment services [105], likely because of a range of factors acting across multiple levels and domains.

At the individual level, limited English language proficiency (socio‐cultural domain) could be a factor affecting health literacy (healthcare domain), and thus the *capability* to seek help, especially when this intersects with discriminatory healthcare practices — such as a lack of non‐English language materials (societal level, healthcare domain). Additionally, policies like the US Affordable Care Act (societal level, behaviour domain) influence the *opportunity* for Latinos to access services, with variations in provisions by state (community level, healthcare domain) [106]. Several factors may also affect their *motivation* to seek help. Language barriers and low health literacy can influence the quality of provider–client interactions (interpersonal level, healthcare domain), while alcohol use may be stigmatized for women in Mexican and Central American cultures, leading to internalized discrimination (individual level, power domain) [107]. Further, undocumented Latina migrants may avoid treatment programmes out of fear of immigration retribution (societal level, sociocultural behaviour and power domains) [107].

### Complexity

Intersectionality aims to understand and analyse complexities in the world, requiring intricate approaches [19]. The IAIF embraces this by examining the multifaceted nature of alcohol inequities, considering the role of interlocking systems of oppression and social constructs, such as power, which are difficult to measure and often overlooked. While acknowledging that this complexity may seem to complicate efforts to address inequities, we argue that it is crucial to consider complexity to deepen analyses and develop effective solutions to alcohol‐related inequities.

### Social inequity

The IAIF helps to understand how alcohol inequities are structured and perpetuated by identifying key factors across multiple domains and levels, drawing on diverse literature. It reflects that social inequities are not inevitable but arise from complex interactions between systems of power and oppression at various levels [19].

### Social justice (practical applications)

Central to intersectionality is critical praxis – reflection and action in the pursuit of social justice. The IAIF is designed to support critical praxis among a broad range of stakeholders working to reduce alcohol inequities, by highlighting overlooked areas and promoting relational, multi‐level approaches. Its practical application will vary depending on the user and their objective. For instance, researchers aiming to foster relational thinking could use the IAIF alongside the ‘ask the other question’ method [108], posing questions like ‘Where is the patriarchy in this?’ when addressing an issue that appears to be racist. A scholar investigating the relationship between racial segregation and alcohol use, for example, might be prompted by the framework to also explore the role of sexism in urban design and how these factors intersect. Alternatively, an organization focused on addressing harmful alcohol consumption within a specific intersectional group could use the IAIF to guide collaborative discussions with that community, identifying the factors most pertinent to them. Table 1 provides guidance on how different user groups might apply the IAIF to inform their practice.

**TABLE 1:** Examples of how different groups may apply the Intersectional Alcohol Inequities Framework (IAIF) to their work.

	How this group may use the IAIF	Supporting questions
**Healthcare providers**	To guide holistic assessment of the multiple levels and domains of influence on a patient’s alcohol consumption and related harm To inform comprehensive patient management by considering factors beyond individual behaviour, such as making appropriate referrals to other services and ensuring culturally sensitive interventions To identify recurring challenges within their patient community and highlight opportunities for advocacy To encourage self‐reflection on their own position of power and privilege, and how this may impact interactions with clients	Which factors, across different domains and levels, are most significantly influencing this person’s alcohol consumption? Which factors are modifiable by healthcare intervention, and which may require referral to external services? What factors might be affecting this patient/patient group’s engagement with the healthcare system, treatment or rehabilitation? How can I mitigate against potential barriers to healthcare engagement/quality for this patient/patient group within the healthcare system? Are any of these factors impacting many patients in my community? How can I raise awareness, address or advocate for this issue? How does my own unique intersectional position impact my interactions with clients? How can I ensure equity in my interactions?
**CBOs** ^**a**^	To collaborate with community members in identifying the factors most influencing alcohol consumption and harm within the community To reflect upon current activities and identify whether any relevant levels or domains have been overlooked To identify areas of unmet need within the community To design multifaceted programmes and interventions	Which levels, domains or specific factors are most relevant to our community? Which levels or domains have we not previously considered? What additional steps can we take to understand these levels or domains within our community? Are we incorporating all critical levels, domains and factors into our interventions? Are there other organizations providing support in these areas or are there any gaps in need within our community?
**Researchers**	To identify gaps in current research by examining overlooked intersectional groups, levels, domains or specific factors To promote the identification of novel, previously unconsidered, research questions To support the consideration of variables beyond the individual (e.g. structural racism or physical alcohol environment) To encourage multi‐level, relational analyses that account for the broad influences on alcohol consumption/harm To identify opportunities for transdisciplinary research, incorporating perspectives from fields such as sociology, public health, medicine, history, behavioural science, biology, political science and economics To help guide rich discussion of study findings and help to understand/explain unexpected findings	What areas does our research and that of our field currently focus on? Are there any intersectional groups, factors, levels or domains that remain understudied? Are we effectively capturing the interactions between different groups, levels and domains in our research design? What questions need answering regarding understudied levels or domains? Are there scholars from other disciplines or departments who could provide fresh perspectives on understudied levels and domains? What is the role of different forms of discrimination (racism, sexism, heterosexism, etc.) in relation to this area of study? Beyond the immediate levels and domains examined in our study, what alternative factors might be influencing our findings? How might the intersectional positions of the research team shape our interpretation of findings? Do we need to engage other stakeholders to gain alternative insights into this issue?
**Public health modelers**	To support the development of conceptual modelling that considers the multi‐level factors influencing alcohol consumption and harm To guide the setting of model boundaries To facilitate the consideration of interactions between social categories, and between social categories and the levels and domains of influence in the model	Are we incorporating variables from all relevant levels and domains in our models? Which of these levels, domains or factors are most critical to model/contribute the most to consumption and/or harm? Which factors are modifiable through intervention/policy? For which factors are data available, and which are feasible to model? Are there factors that are identified as important but are currently unfeasible to model? What changes are needed to enable the modelling of these factors (e.g. changes to routine data collection)? Are we effectively capturing the interactions between social categories (e.g. race, gender or socio‐economic status), levels and domains? How can we simulate the impact of multi‐level interactions on alcohol consumption and related harms?
**Research funders**	To map out current research activities, identifying which groups are being studied and at which levels and domains To identify areas of research need, focusing on overlooked levels and domains, and to encourage proposals that incorporate these aspects To identify projects that address multi‐level and intersectional factors influencing alcohol‐related harm	Are we funding research that captures the complexity of alcohol consumption and harm across the various levels and domains? Are there underfunded areas that need more attention? How can we ensure our funding priorities address intersectional disparities in alcohol‐related outcomes?
**Policymakers**	To identify gaps in current policies and practice To design policies that address multiple levels and domains of influence To identify areas for cross‐sector collaboration, such as between the Department of Health and Human Services (healthcare domain) and the Department of Housing and Urban Development (physical environment domain)	Are we considering all pertinent levels and domains when shaping alcohol‐related policies? Are there any intersectional groups that may be overlooked by our policies? Who else could we collaborate with to ensure a comprehensive, multifactorial approach to addressing alcohol‐related harms and inequities?

^a^ CBO, community‐based organization.

## DISCUSSION

We propose the IAIF, a conceptual framework that visually illustrates many pertinent factors influencing alcohol consumption, harm and inequities, across five levels and seven domains. Drawing upon the key tenets of intersectionality, we further develop the NIMHD framework by adding a ‘power’ domain and a ‘historical’ level to capture a more comprehensive set of factors, and stress the importance of context and relationality. We also incorporate a ‘digital environment’ domain, to reflect an important element of contemporary social context, as suggested by Richardson *et al*. [30]. Though developed for the context of the USA, its structure allows for application in diverse contexts, as discussed in its practical applications and adaptability.

### Generalizability

In this version of the IAIF, we present factors relevant to the US context. However, many of these factors are intentionally broad — for example, ‘community norms’ — to facilitate utilization across diverse contexts. We believe the broad domains and levels should provide a useful starting point for any context and that the framework could be systematically adapted to other settings, such as another country, by removing, adding or adapting specific factors, where relevant. For instance, in the context of the UK, the National Health Service provides publicly funded healthcare based on clinical need rather than ability to pay. The factor ‘individual health insurance’ may therefore be considered less relevant.

Additionally, the IAIF could be adapted to reflect the factors pertinent to specific groups within a given context. For example, a community‐based organization (CBO) may use the framework to identify the factors most relevant to a particular intersectional group in their community. Engagement with people with lived experience and intra‐categorical studies, which examine complexities within specific intersectional groups, would be beneficial in such instances.

Lastly, while the IAIF is designed to be comprehensive regarding alcohol use and related harms, many of the identified factors are likely to be applicable to broader substance use and other public health behaviours. Conducting case studies that apply the IAIF in these areas would be valuable.

### Limitations

There are several limitations in the methods used to develop this framework. First, this initial iteration of the IAIF was developed solely by researchers. Input from a broader range of stakeholders, including people with lived experience, CBOs, clinicians and policymakers, could have provided additional insights.

We consider the lack of input from people with lived experience to be a key limitation of the current iteration of the framework. Although our research group includes at least one author with lived experience of a close family member affected by AUD, broader and more diverse lived experience perspectives are needed. As people with lived experience constitute a heterogeneous group, we envision that the framework could be presented to specific communities by local CBOs that work with them, particularly in the context of developing community‐tailored interventions. This process could ensure reciprocal benefit and yield: (i) targeted adaptations of the framework for the given population; and (ii) the identification of broader factors, levels and domains that may be applicable across populations. We hope such engagement can be pursued in future research, whether by our team or by others with established community partnerships.

Similarly, our research group lacks clinicians with substantial experience of working directly with individuals affected by AUD. Clinician input may provide nuanced insights into the healthcare domain, such as regarding patient (dis)engagement, and the influence of institutional policies, clinical biases, etc. Clinicians are therefore another key stakeholder group to whom the IAIF should be presented for future refinement.

Further, while the authorship is diverse in terms of gender, race, ethnicity and geographical location, there is less variability in terms of education level and SES (the author characteristics are provided in Appendix S2). Although we have engaged in reflexivity — considering our social positions, roles and access to power — it is inevitable that these factors have influenced the development of the IAIF.

Second, we acknowledge that the framework has yet to be practically applied. We anticipate that this core version will be further refined through real‐world application, and we welcome critique, enhancement and adaptation from others. As aforementioned, we foresee specific adaptations of the framework to tailor it to particular groups and contexts, as demonstrated with the original NIMHD framework [109].

Third, whilst we supplemented the group discussions with literature searching, this was done via targeted searches and citation chaining, rather than using a systematic search strategy. However, to support a thorough understanding of the factors at play, we have consulted existing evidence reviews and models, such as that published by Gell *et al*. in 2016 [110], and have drawn upon best‐practice guidance on the development of conceptual models in Public Health [111].

Finally, while the broad components of the framework should allow for systematic adaptation to different settings, it is important to note that the evidence supporting these components primarily comes from the Global North. Adapting the framework to the Global South would require cultivating dialogue with scholars and activists within those contexts, and context‐specific testing. Such efforts would offer a richer understanding of the global complexity of alcohol‐related inequities.

## CONCLUSION

To our knowledge, the IAIF is the first comprehensive intersectional representation of factors influencing alcohol inequities. We believe that adding an intersectional lens to the existing NIMHD research framework, specifically considering the historical context, the influence of power and relationality, provides a more comprehensive understanding of complex public health problems. We foresee several practical applications of the IAIF in relation to both research and critical praxis and provide examples of how a variety of actors may apply the framework to their work. The framework may be adapted to reflect alternative public health problems and contexts.

## AUTHOR CONTRIBUTIONS

**Sophie Bright:** Conceptualization (lead); data curation (lead); formal analysis (lead); methodology (lead); project administration (lead); software (lead); visualization (lead); writing—original draft (lead). **Charlotte Buckley:** Conceptualization (supporting); formal analysis (supporting); methodology (supporting); project administration (supporting); supervision (supporting); visualization (equal); writing—review and editing (supporting). **Daniel Holman:** Conceptualization (supporting); formal analysis (supporting); methodology (supporting); project administration (supporting); supervision (equal); visualization (supporting); writing—review and editing (supporting). **Hazel Squires:** Conceptualization (supporting); methodology (supporting); visualization (supporting); writing—review and editing (supporting). **Naomi Greene:** Conceptualization (supporting); visualization (supporting); writing—review and editing (supporting). **Nina Mulia:** Conceptualization (supporting); visualization (supporting); writing—review and editing (supporting). **Carolin Kilian:** Conceptualization (supporting); visualization (supporting); writing—review and editing (supporting). **Charlotte Probst:** Conceptualization (supporting); visualization (supporting); writing—review and editing (supporting). **Colin Angus:** Conceptualization (supporting); visualization (supporting); writing—review and editing (supporting). **John Holmes:** Conceptualization (supporting); visualization (supporting); writing—review and editing (supporting). **Helena M. Constante:** Conceptualization (supporting); visualization (supporting); writing—review and editing (supporting). **Meesha Warmington:** Conceptualization (supporting); visualization (supporting); writing—review and editing (supporting). **Robin Purshouse:** Conceptualization (supporting); formal analysis (supporting); methodology (supporting); project administration (supporting); supervision (equal); visualization (supporting); writing—review and editing (supporting).

## DECLARATION OF INTERESTS

None.

## Supporting information


**Appendix S1.** Supporting information.

**Appendix S2.** Supporting Information.

## Data Availability

Data sharing not applicable to this article as no datasets were generated or analysed during the current study.

## References

[1] Rehm J , Gmel GE , Gmel G , Hasan OSM , Imtiaz S , Popova S , et al. The relationship between different dimensions of alcohol use and the burden of disease‐an update. Addiction. 2017;112(6):968–1001. 28220587 10.1111/add.13757 PMC5434904

[2] World Health Organization . Alcohol [Internet]. 2024 [cited 2024 Dec 26]. Available from: https://www.who.int/news-room/fact-sheets/detail/alcohol

[3] World Health Organization . Health inequities and their causes [Internet]. 2018 [cited 2024 Dec 26]. Available from: https://www.who.int/news-room/facts-in-pictures/detail/health-inequities-and-their-causes

[4] Chartier K , Caetano R . Ethnicity and health disparities in alcohol research. Alcohol Res Health. 2010;33(1–2):152–160. 21209793 PMC3887493

[5] Delker E , Brown Q , Hasin DS . Alcohol consumption in demographic subpopulations. Alcohol Res. 2016;38(1):7–15. 27159807 10.35946/arcr.v38.1.02 PMC4872616

[6] Mulia N , Ye Y , Greenfield TK , Zemore SE . Disparities in alcohol‐related problems among White, Black and Hispanic Americans. Alcohol Clin Exp Res. 2009;33(4):654–662. 10.1111/j.1530-0277.2008.00880.x 19183131 PMC2771773

[7] Mulia N , Karriker‐Jaffe KJ , Witbrodt J , Bond J , Williams E , Zemore SE . Racial/ethnic differences in 30‐year trajectories of heavy drinking in a nationally representative U.S. sample. Drug Alcohol Depend. 2017;170:133–141. 27889594 10.1016/j.drugalcdep.2016.10.031 PMC5270645

[8] Probst C , Lange S , Kilian C , Saul C , Rehm J . The dose‐response relationship between socioeconomic deprivation and alcohol‐attributable mortality risk—a systematic review and meta‐analysis. BMC Med. 2021;19(1):268. 10.1186/s12916-021-02132-z 34736475 PMC8569998

[9] White AM . Gender differences in the epidemiology of alcohol use and related harms in the United States. Alcohol Res. 2020;40(2):01. 10.35946/arcr.v40.2.01 PMC7590834 33133878

[10] Witbrodt J , Mulia N , Zemore SE , Kerr WC . Racial/ethnic disparities in alcohol‐related problems: differences by gender and level of heavy drinking. Alcohol Clin Exp Res. 2014;38(6):1662–1670. 10.1111/acer.12398 24730475 PMC4047188

[11] Xu Y , Geldsetzer P , Manne‐Goehler J , Theilmann M , Marcus ME , Zhumadilov Z , et al. The socioeconomic gradient of alcohol use: an analysis of nationally representative survey data from 55 low‐income and middle‐income countries. Lancet Glob Health. 2022;10(9):e1268–e1280. 10.1016/S2214-109X(22)00273-X 35961350 PMC9582994

[12] Collins PH . It's all in the family: intersections of gender, race, and nation. Hypatia. 1998;13(3):62–82. 10.1111/j.1527-2001.1998.tb01370.x

[13] Combahee River Collective . The Combahee River Collective Statement. In: Smith B , editorHome girls: a Black feminist anthology Kitchen Table: Women of Color Press; 1977. p. 272–282.

[14] Crenshaw K . Demarginalizing the intersection of race and sex: A Black feminist critique of antidiscrimination doctrine, feminist theory and antiracist politics. Univ Chic Leg Forum. 1989;1989(1) Article 8:139–167.

[15] Collins PH . Black feminist thought: Knowledge, consciousness, and the politics of empowerment 1st ed. Routledge Classics; 1991.

[16] Bauer GR . Incorporating intersectionality theory into population health research methodology: Challenges and the potential to advance health equity. Soc Sci Med. 2014;110:10–17. 24704889 10.1016/j.socscimed.2014.03.022

[17] Bowleg L . The problem with the phrase women and minorities: intersectionality‐an important theoretical framework for public health. Am J Public Health. 2012;102(7):1267–1273. 22594719 10.2105/AJPH.2012.300750 PMC3477987

[18] Kapilashrami A , Hankivsky O . Intersectionality and why it matters to global health. Lancet. 2018;391(10140):2589–2591. 10.1016/S0140-6736(18)31431-4 30070211

[19] Collins PH , Bilge S . Intersectionality 2nd ed. Cambridge, UK: Polity Press; 2020.

[20] Brady SS , Brubaker L , Fok CS , Gahagan S , Lewis CE , Lewis J , et al. Development of conceptual models to guide public health research, practice, and policy: synthesizing traditional and contemporary paradigms. Health Promot Pract. 2020;21(4):510–524. Available from: 10.1177/1524839919890869 31910039 PMC7869957

[21] NIMHD . NIMHD minority health and health disparities research framework [Internet]. 2024 [cited 2024 Jan 26]. Available from: https://www.nimhd.nih.gov/about/overview/research-framework/nimhd-framework.html

[22] Hill CV , Pérez‐Stable EJ , Anderson NA , Bernard MA . The National Institute on Aging health disparities research framework. Ethn Dis. 2015;25(3):245–254. 10.18865/ed.25.3.245 26675362 PMC4671408

[23] Bronfenbrenner U . Toward an experimental ecology of human development. Am Psychol. 1977;32(7):513–531. 10.1037/0003-066X.32.7.513

[24] NIMHD . NIMHD minority health and health disparities research framework [Internet]. 2023 [cited 2024 Jan 28]. Available from: https://www.nimhd.nih.gov/about/overview/research-framework/

[25] Buckley C , Ye Y , Kerr WC , Mulia N , Puka K , Rehm J , et al. Trends in mortality from alcohol, opioid, and combined alcohol and opioid poisonings by sex, educational attainment, and race and ethnicity for the United States 2000–2019. BMC Med. 2022;20(1):405. 10.1186/s12916-022-02590-z 36280833 PMC9590383

[26] Matarazzo A , Hennekens CH , Dunn J , Benson K , Willett Y , Levine RS , et al. New clinical and public health challenges: increasing trends in United States alcohol related mortality. Am J Med. 2024;138(3):477–486. Available from: https://www.amjmed.com/article/S0002-9343(24)00704-6/abstract 39532247 10.1016/j.amjmed.2024.10.024

[27] Probst C , Könen M , Rehm J , Sudharsanan N . Alcohol‐attributable deaths help drive growing socioeconomic inequalities in US life expectancy, 2000‐18. Health Aff (Millwood). 2022;41(8):1160–1168. 10.1377/hlthaff.2021.01905 35914205 PMC9639704

[28] Alvidrez J , Castille D , Laude‐Sharp M , Rosario A , Tabor D . The National Institute on Minority Health and Health Disparities research framework. Am J Public Health. 2019;109(S1):S16–S20. 10.2105/AJPH.2018.304883 30699025 PMC6356129

[29] Haley S , Jardine S , Kelvin E , Herrmann C , Maroko A . Neighborhood alcohol outlet density, historical redlining, and violent crime in NYC 2014–2018. Int J Environ Res Public Health. 2023;20(4):3212. 36833907 10.3390/ijerph20043212 PMC9963869

[30] Richardson S , Lawrence K , Schoenthaler AM , Mann D . A framework for digital health equity. npj Digital Med. 2022;5(1):1–6. 10.1038/s41746-022-00663-0 PMC9387425 35982146

[31] Collins PH . Black feminist thought: Knowledge, consciousness, and the politics of empowerment Psychology Press; 2000 353 p.

[32] Collins PH . The difference that power makes: Intersectionality and participatory democracy. In: Hankivsky O , Jordan‐Zachery JS , editorsThe palgrave handbook of intersectionality in public policy [internet] Cham: Springer International Publishing; 2019. p. 167–192 Available from: 10.1007/978-3-319-98473-5_7.

[33] Schmidt LA . The equal right to drink. Drug Alcohol Rev. 2014;33(6):581–587. 10.1111/dar.12215 25303360

[34] James D . Health and health‐related correlates of internalized racism among racial/ethnic minorities: a review of the literature. J Racial Ethn Health Disparities. 2020;7(4):785–806. 10.1007/s40615-020-00726-6 32086794

[35] Corrigan P , Schomerus G , Shuman V , Kraus D , Perlick D , Harnish A , et al. Developing a research agenda for understanding the stigma of addictions part I: lessons from the mental health stigma literature. Am J Addict. 2017;26(1):59–66. 10.1111/ajad.12458 27779803

[36] O'Donnell AT , Foran AM . The link between anticipated and internalized stigma and depression: A systematic review. Soc Sci Med. 2024;349:116869. 38678910 10.1016/j.socscimed.2024.116869

[37] Keyes KM , Hatzenbuehler ML , McLaughlin KA , Link B , Olfson M , Grant BF , et al. Stigma and treatment for alcohol disorders in the United States. Am J Epidemiol. 2010;172(12):1364–1372. 21044992 10.1093/aje/kwq304 PMC2998202

[38] Markman Geisner I , Larimer ME , Neighbors C . The relationship among alcohol use, related problems, and symptoms of psychological distress: gender as a moderator in a college sample. Addict Behav. 2004;29(5):843–848. 10.1016/j.addbeh.2004.02.024 15219328

[39] Turner S , Mota N , Bolton J , Sareen J . Self‐medication with alcohol or drugs for mood and anxiety disorders: a narrative review of the epidemiological literature. Depress Anxiety. 2018;35(9):851–860. 10.1002/da.22771 29999576 PMC6175215

[40] Jemal A , Gunn A , Inyang C . Transforming responses: Exploring the treatment of substance‐using African‐American women. J Ethn Subst Abuse. 2019;19(4):659. 30940008 10.1080/15332640.2019.1579141 PMC6776726

[41] Watson RJ , Fish JN , Poteat VP , Rathus T . Sexual and gender minority youth alcohol use: within‐group differences in associations with internalized stigma and victimization. J Youth Adolesc. 2019;48(12):2403–2417. 10.1007/s10964-019-01130-y 31605292 PMC6872943

[42] Hughes TL , Wilsnack SC , Kantor LW . The influence of gender and sexual orientation on alcohol use and alcohol‐related problems. Alcohol Res. 2016;38(1):121–132. 27159819 10.35946/arcr.v38.1.15 PMC4872607

[43] Rolfe A , Orford J , Dalton S . Women, alcohol and femininity: A discourse analysis of women heavy drinkers' accounts. J Health Psychol. 2009;14(2):326–335. 10.1177/1359105308100217 19237500

[44] Room R . Stigma, social inequality and alcohol and drug use. Drug Alcohol Rev. 2005;24(2):143–155. 10.1080/09595230500102434 16076584

[45] Castro FG , Stein JA , Bentler PM . Ethnic pride, traditional family values, and acculturation in early cigarette and alcohol use among Latino adolescents. J Prim Prev. 2009;30(3–4):265–292. 10.1007/s10935-009-0174-z 19415497 PMC2818880

[46] Woo B , Fan W , Tran TV , Takeuchi DT . The role of racial/ethnic identity in the association between racial discrimination and psychiatric disorders: A buffer or exacerbator? SSM ‐ Population Health. 2019;7:100378. 30923732 10.1016/j.ssmph.2019.100378 PMC6423488

[47] Nelson AR , Stith AY , Smedley BD (Eds). Unequal treatment: Confronting racial and ethnic disparities in health care 1st ed. Washington, D.C: National Academies Press; 2002 780 p. 25032386

[48] Cromwell RE , Olsen DH (Eds). Power in families Oxford, England: Sage; 1975. p. xvii–264.

[49] Wilson IM , Willoughby B , Tanyos A , Graham K , Walker M , Laslett AM , et al. A global review of the impact on women from men's alcohol drinking: the need for responding with a gendered lens. Glob Health Action. 2024;17(1):2341522. 10.1080/16549716.2024.2341522 38700277 PMC11073422

[50] Foran HM , O'Leary KD . Alcohol and intimate partner violence: a meta‐analytic review. Clin Psychol Rev. 2008;28(7):1222–1234. 10.1016/j.cpr.2008.05.001 18550239

[51] White HR , Chen PH . Problem drinking and intimate partner violence. J Stud Alcohol. 2002;63(2):205–214. 10.15288/jsa.2002.63.205 12033697

[52] Laslett AM , Stanesby O , Graham K , Callinan S , Karriker‐Jaffe KJ , Wilsnack S , et al. Children's experience of physical harms and exposure to family violence from others' drinking in nine societies. Addict Res Theor. 2020;28(4):354–364. 10.1080/16066359.2019.1704272 PMC7591104 33122974

[53] Nayak MB , Patterson D , Wilsnack SC , Karriker‐Jaffe KJ , Greenfield TK . Alcohol's secondhand harms in the United States: new data on prevalence and risk factors. J Stud Alcohol Drugs. 2019;80(3):273–281. 10.15288/jsad.2019.80.273 31250790 PMC6614929

[54] Karriker‐Jaffe KJ , Greenfield TK , Kaplan LM . Distress and alcohol‐related harms from intimates, friends, and strangers. J Subst Abuse. 2016;22(4):434. 10.1080/14659891.2016.1232761 PMC5530071 28757806

[55] Gilens M . Affluence and influence: Economic inequality and political power in America [internet] Princeton University Press; 2012.

[56] Perrin PB , Sutter ME , Trujillo MA , Henry RS , Pugh M Jr . The minority strengths model: development and initial path analytic validation in racially/ethnically diverse LGBTQ individuals. J Clin Psychol. 2020;76(1):118–136. 10.1002/jclp.22850 31468539 PMC6908758

[57] Bryden A , Roberts B , Petticrew M , McKee M . A systematic review of the influence of community level social factors on alcohol use. Health Place. 2013;21:70–85. 10.1016/j.healthplace.2013.01.012 23454663

[58] Garcia V , Lambert E , Fox K , Heckert D , Pinchi NH . Grassroots interventions for alcohol use disorders in the Mexican immigrant community: a narrative literature review. J Ethn Subst Abuse. 2022;21(3):773–792. 10.1080/15332640.2020.1803781 32757884 PMC8245010

[59] Lundgren L , Amodeo M , Cohen A , Chassler D , Horowitz A . Modifications of evidence‐based practices in community‐based addiction treatment organizations: a qualitative research study. Addict Behav. 2011;36(6):630–635. 10.1016/j.addbeh.2011.01.003 21310541

[60] Substance Abuse and Mental Health Services Administration (SAMHSA) . Community engagement: An essential component of an effective and equitable substance use prevention system|SAMHSA publications and digital products [internet] Rockville, MD: National Mental Health and Substance Use Policy Laboratory; 2022 [cited 2024 Oct 27]. Report No.: PEP22‐06‐01–005. Available from: https://store.samhsa.gov/product/community‐engagement‐essential‐component‐effective‐and‐equitable‐substance‐use‐prevention

[61] Wilson RT , Till BD . Targeting of outdoor alcohol advertising: a study across ethnic and income groups. J Curr Issues Res Advertising. 2012;33(2):267–281. 10.1080/10641734.2012.700800

[62] Moore H , Jones‐Webb R , Toomey T , Lenk K . Alcohol advertising on billboards, transit shelters, and bus benches in Inner‐City neighborhoods. Contemp Drug Probl. 2008;35(2–3):509–532. 10.1177/009145090803500214

[63] Evans‐Polce RJ , Jang BJ , Maggs JL , Patrick ME . Gender and age differences in the associations between family social roles and excessive alcohol use. Soc Sci Med. 2020;244:112664. 31726267 10.1016/j.socscimed.2019.112664 PMC6983322

[64] Hatzenbuehler ML , McLaughlin KA , Keyes KM , Hasin DS . The impact of institutional discrimination on psychiatric disorders in lesbian, gay, and bisexual populations: A prospective study. Am J Public Health. 2010;100(3):452–459. 10.2105/AJPH.2009.168815 20075314 PMC2820062

[65] Black Lives Matter, Black Lives Matter Global Network Foundation . Black Lives Matter. 2024 [cited 2024 Jun 3]. Black lives Matter. Available from: https://blacklivesmatter.com/about/

[66] Eichstaedt JC , Sherman GT , Giorgi S , Roberts SO , Reynolds ME , Ungar LH , et al. The emotional and mental health impact of the murder of George Floyd on the US population. Proc Natl Acad Sci U S A. 2021;118(39):e2109139118. 10.1073/pnas.2109139118 34544875 PMC8488615

[67] Curtis DS , Washburn T , Lee H , Smith KR , Kim J , Martz CD , et al. Highly public anti‐Black violence is associated with poor mental health days for Black Americans. Proc Natl Acad Sci. 2021;118(17):e2019624118. 10.1073/pnas.2019624118 33875593 PMC8092615

[68] Brannon TN . Racism hurts, can antiracism heal?: Positive mental health correlates of antiracist engagement. PNAS Nexus. 2023;2(10):pgad309. 37799326 10.1093/pnasnexus/pgad309 PMC10548497

[69] Baskin‐Sommers A , Simmons C , Conley M , Chang SA , Estrada S , Collins M , et al. Adolescent civic engagement: lessons from Black lives Matter. Proc Natl Acad Sci. 2021;118(41):e2109860118. 10.1073/pnas.2109860118 34607958 PMC8521674

[70] Field A , Park CY , Theophilo A , Watson‐Daniels J , Tsvetkov Y . An analysis of emotions and the prominence of positivity in #BlackLivesMatter tweets. Proc Natl Acad Sci. 2022;119(35):e2205767119. 10.1073/pnas.2205767119 35998217 PMC9436370

[71] Ni MY , Kim Y , McDowell I , Wong S , Qiu H , Wong IO , et al. Mental health during and after protests, riots and revolutions: A systematic review. Aust N Z J Psychiatry. 2020;54(3):232–243. 10.1177/0004867419899165 31989834

[72] Popova S , Giesbrecht N , Bekmuradov D , Patra J . Hours and days of Sale and density of alcohol outlets: impacts on alcohol consumption and damage: a systematic review. Alcohol Alcohol. 2009;44(5):500–516. 10.1093/alcalc/agp054 19734159

[73] Sherk A , Stockwell T , Chikritzhs T , Andréasson S , Angus C , Gripenberg J , et al. Alcohol consumption and the physical availability of take‐away alcohol: systematic reviews and Meta‐analyses of the days and hours of Sale and outlet density. J Stud Alcohol Drugs. 2018;79(1):58–67. 10.15288/jsad.2018.79.58 29227232

[74] Han D , Gorman DM . Socio‐spatial patterning of off‐sale and on‐sale alcohol outlets in a Texas city. Drug Alcohol Rev. 2014;33(2):152–160. 10.1111/dar.12096 24320205 PMC3951209

[75] Lee JP , Ponicki W , Mair C , Gruenewald P , Ghanem L . What explains the concentration of off‐premise alcohol outlets in Black neighborhoods? SSM Popul Health. 2020;12:100669. 10.1016/j.ssmph.2020.100669 33102679 PMC7576518

[76] McKinzie AE , Richards PL . An argument for context‐driven intersectionality. Sociol Compass. 2019;13(4):e12671. 10.1111/soc4.12671

[77] Yellow Horse Brave Heart M . The historical trauma response among natives and its relationship with substance abuse: A Lakota illustration. J Psychoactive Drugs. 2003;35(1):7–13. 10.1080/02791072.2003.10399988 12733753

[78] Gone JP , Hartmann WE , Pomerville A , Wendt DC , Klem SH , Burrage RL . The impact of historical trauma on health outcomes for indigenous populations in the USA and Canada: a systematic review. Am Psychol. 2019;74(1):20–35. 10.1037/amp0000338 30652897

[79] McNiece ZP . Ripples in sand and time: Intergenerational trauma in queer communities [Internet] [Ph.D.] United States ‐‐ Florida: University of Florida; 2022 [cited 2024 Oct 30]. Available from: https://www.proquest.com/docview/2830019409/abstract/213E49CC2D1842A5PQ/1

[80] Washington H . Medical apartheid. The dark history of medical experimentation on Black Americans from colonial times to the present Knopf Doubleday Publishing Group; 2008.

[81] Hoffman KM , Trawalter S , Axt JR , Oliver MN . Racial bias in pain assessment and treatment recommendations, and false beliefs about biological differences between blacks and whites. Proc Natl Acad Sci U S A. 2016;113(16):4296–4301. 10.1073/pnas.1516047113 27044069 PMC4843483

[82] Olfson M , Zuvekas SH , McClellan C , Wall MM , Hankerson SH , Blanco C . Racial‐ethnic disparities in outpatient mental health Care in the United States. PS. 2023;74(7):674–683. 10.1176/appi.ps.20220365 36597696

[83] Zale EL , Maisto SA , Ditre JW . Interrelations between pain and alcohol: an integrative review. Clin Psychol Rev. 2015;37:57–71. 10.1016/j.cpr.2015.02.005 25766100 PMC4385458

[84] Louie P , Wilkes R . Representations of race and skin tone in medical textbook imagery. Soc Sci Med. 2018 Apr;202:38–42. 10.1016/j.socscimed.2018.02.023 29501717

[85] Hilbert M . Digital technology and social change: the digital transformation of society from a historical perspective. Dialogues Clin Neurosci. 2020;22(2):189–194. 10.31887/DCNS.2020.22.2/mhilbert 32699519 PMC7366943

[86] Riper H , Hoogendoorn A , Cuijpers P , Karyotaki E , Boumparis N , Mira A , et al. Effectiveness and treatment moderators of internet interventions for adult problem drinking: an individual patient data meta‐analysis of 19 randomised controlled trials. PLoS Med. 2018;15(12):e1002714. 10.1371/journal.pmed.1002714 30562347 PMC6298657

[87] Hegeman PC , Vader DT , Kamke K , El‐Toukhy S . Patterns of digital health access and use among US adults: a latent class analysis. BMC Digital Health. 2024;2(1):42. 10.1186/s44247-024-00100-0 39544227 PMC11562959

[88] Kontos E , Blake KD , Chou WYS , Prestin A . Predictors of eHealth usage: insights on the digital divide from the health information National Trends Survey 2012. J Med Internet Res. 2014;16(7):e172. 10.2196/jmir.3117 25048379 PMC4129114

[89] Roy‐Highley E , Körner K , Mulrenan C , Petticrew M . Dark patterns, dark nudges, sludge and misinformation: Alcohol industry apps and digital tools. Health Promot Int. 2024;39(5):daae037. 39377424 10.1093/heapro/daae037

[90] Carah N , Brodmerkel S . Alcohol Marketing in the era of digital media platforms. J Stud Alcohol Drugs. 2021;82(1):18–27. 10.15288/jsad.2021.82.18 33573719

[91] Noel JK , Sammartino CJ , Rosenthal SR . Exposure to digital alcohol marketing and alcohol use: a systematic review. J Stud Alcohol Drugs Suppl. 2020;(s19):57–67. 10.15288/jsads.2020.s19.57 32079562 PMC7064004

[92] Lobstein T , Landon J , Thornton N , Jernigan D . The commercial use of digital media to market alcohol products: a narrative review. Addiction. 2017;112(Suppl 1):21–27. 10.1111/add.13493 27327239

[93] Zhang L , Esser MB . U.S. adolescents' exposure to alcohol marketing: Self‐reported exposure on the internet and traditional media. AJPM Focus. 2024;3(5):100243. Available from: https://www.ajpmfocus.org/article/S2773-0654(24)00061-0/fulltext 39430134 10.1016/j.focus.2024.100243 PMC11486924

[94] D'Amico EJ , Martino SC , Collins RL , Shadel WG , Tolpadi A , Kovalchik S , et al. Factors associated with younger adolescents' exposure to online alcohol advertising. Psychol Addict Behav. 2017;31(2):212–219. 10.1037/adb0000224 27819430 PMC5344756

[95] Karlamangla A , Zhou K , Reuben D , Greendale G , Moore A . Longitudinal trajectories of heavy drinking in adults in the United States of America. Addiction. 2006;101(1):91–99. 10.1111/j.1360-0443.2005.01299.x 16393195

[96] Lui CK , Kerr WC , Mulia N , Ye Y . Educational differences in alcohol consumption and heavy drinking: An age‐period‐cohort perspective. Drug Alcohol Depend. 2018;186:36–43. 29544120 10.1016/j.drugalcdep.2017.12.046 PMC6003414

[97] Probst C , Kilian C , Sanchez S , Lange S , Rehm J . The role of alcohol use and drinking patterns in socioeconomic inequalities in mortality: a systematic review. Lancet Public Health. 2020;5(6):e324–e332. 10.1016/S2468-2667(20)30052-9 32504585

[98] NIAAA . Alcohol use in the United States: Age groups and demographic characteristics|National Institute on Alcohol Abuse and Alcoholism (NIAAA) [Internet]. 2024 [cited 2024 Jul 8]. Available from: https://www.niaaa.nih.gov/alcohols-effects-health/alcohol-topics/alcohol-facts-and-statistics/alcohol-use-united-states-age-groups-and-demographic-characteristics

[99] Chartier K , Vaeth P , Caetano R . Focus on: Ethnicity and the social and health harms from drinking. Alcohol Res. 2014;35(2):229–237. 10.35946/arcr.v35.2.13 PMC3908714 24881331

[100] Bright S , Buckley C , Holman D , Leckie G , Bell A , Mulia N , et al. An analysis of intersectional disparities in alcohol consumption in the US. Soc Sci Med. 2024;363:117514. 10.1016/j.socscimed.2024.117514 39566226 PMC12857125

[101] Greene N , Jackson JW , Dean LT . Examining disparities in excessive alcohol use among Black and Hispanic lesbian and bisexual women in the United States: An intersectional analysis. J Stud Alcohol Drugs. 2020;81(4):462–470. 32800082 10.15288/jsad.2020.81.462 PMC7437553

[102] Parthasarathy S , Chi FW , Metz V , Kline‐Simon A , Asyyed A , Campbell CI , et al. Disparities in the receipt of alcohol brief intervention: the intersectionality of sex, age, and race/ethnicity. Addiction. 2023;118(7):1258–1269. 10.1111/add.16195 36988614

[103] Wagner FA , Cano M , Kamugisha S . The intersectionality framework is a powerful tool to understand underage alcohol use. J Adolesc Health. 2024;75(2):207–208. 10.1016/j.jadohealth.2024.05.001 39025586

[104] Michie S , van Stralen MM , West R . The behaviour change wheel: a new method for characterising and designing behaviour change interventions. Implement Sci. 2011;6(1):42. 10.1186/1748-5908-6-42 21513547 PMC3096582

[105] Zemore SE , Murphy RD , Mulia N , Gilbert PA , Martinez P , Bond J , et al. A moderating role for gender in racial/ethnic disparities in alcohol services utilization: results from the 2000 to 2010 national alcohol surveys. Alcohol Clin Exp Res. 2014;38(8):2286–2296. 10.1111/acer.12500 25041173 PMC4146628

[106] Alcalá HE , Chen J , Langellier BA , Roby DH , Ortega AN . Impact of the affordable care act on health care access and utilization among Latinos. J Am Board Fam Med. 2017;30(1):52–62. 10.3122/jabfm.2017.01.160208 28062817

[107] Pagano A . Barriers to drug abuse treatment for LATINO migrants: treatment providers' perspectives. J Ethn Subst Abuse. 2014;13(3):273–287. 10.1080/15332640.2014.886320 25176120 PMC4388552

[108] Matsuda MJ . Beside my sister, facing the enemy: legal theory out of coalition. Stanford Law Rev. 1991;43(6):1183–1192. 10.2307/1229035

[109] Lafarga Previdi I , Vélez Vega CM . Health disparities research framework adaptation to reflect Puerto Rico's socio‐cultural context. Int J Environ Res Public Health. 2020;17(22):8544. 10.3390/ijerph17228544 33217956 PMC7698747

[110] Gell L , Bühringer G , Mcleod J , Forberger S , Holmes J , Lingford‐Hughes A , Meier PS What determines harm from addictive substances and Behaviours? 2016. 10.1093/acprof:oso/9780198746683.001.0001

[111] Squires H , Chilcott J , Akehurst R , Burr J , Kelly MP . A framework for developing the structure of public health economic models. Value Health. 2016;19(5):588–601. 10.1016/j.jval.2016.02.011 27565276

