# Supplementary material for: Applying an intersectional lens to alcohol inequities: A conceptual framework
Source: Addiction. 2025 Jul 14;120(12):2585–98. doi: 10.1111/add.70130 (PMC12586755; doi:10.1111/add.70130)
Supplement: Supplementary file 1 — Appendix S1. Supporting information. [file ADD-120-2585-s002.pdf]

Appendix 1 – Tables showing additional detail for each IAIF domain

		DOMAIN OF INFLUENCE
		**POWER**
-	**Individual**	**Intersecting socially constructed factors (race, gender, socioeconomic status etc.)**<br>- Social factors related with levels of consumption and/or the relationship between consumption and harm, e.g. increased consumption associated (in broad, unitary terms) with younger age (18-34), male gender, non-Hispanic White race, higher household income, sexual minority status, lower public religiosity, and single status; disproportionately increased harm associated (in broad unitary terms) with low SES, Native American, Black, and Hispanic race and ethnicity (1)<br>- Social factors overlap and interact e.g., interaction noted among sexual orientation/gender/race, and between race/age ("crossover" effect)<br>**Internalised discrimination and self-stigma**<br>- In relation to socially prescribed categories (such as race, sexual orientation) and/or alcohol use (2,3)<br>**Individual freedom/ rights**<br>- E.g., An individuals access to independent finances, personal autonomy, right to work/education<br>**Racial/ethnic/cultural pride**<br>- Individual's sense of cultural, racial, or ethnic pride can influence alcohol consumption, both directly and indirectly, with effects varying across different social groups (e.g. see Castro et al., 2009; Opara et al., 2023)
	**Interpersonal**	- **Power dynamics within intimate relationships:** Differential power dynamics exist within heterosexual relationships due to patriarchal and gendered social norms, resulting in differences in drinking rates differ by gender, and different forms of harm resulting from harmful drinking (predominately men's harmful drinking)<br>- **Power dynamics during daily interactions:** Power influences interactions between an individual and their peers, colleagues, service providers etc. For example, interactions with healthcare providers influenced by cultural health capital (CHC) (6)
	**Community**	**Level of political leverage**<br>- Affluent, privileged communities often possess greater political leverage than low-income neighbourhoods (7) which can be leveraged to attract resources and drive change, such as lobbying against unwanted alcohol outlets.<br>**Community-based organizations / Community-based participatory research**<br>- For example, Recovery Community Organizations (8)<br>- can support substance use prevention and treatment, by ensuring interventions are tailored to meet the specific needs of communities (Substance Abuse and Mental Health Services Administration (SAMHSA), 2022)<br>**Social capital and community strength**<br>- Social support and community consciousness within minoritized populations can increase identity pride, resilience, and self-esteem, in turn promoting positive health behaviours.<br>**Grassroots movements**<br>- Grassroots movements - treatment modalities that emerge organically from the community, rather than from formal medical institutions - are intrinsically rooted in local culture, making them both more familiar and accessible to the populations they serve (for example, see Garcia et al., 2022)
	**Societal**	**Systems of social power and domination**<br>- **E.g. Patriarchy and Capitalism**<br>**Collective action/ social movements**<br>- e.g., Black Lives Matter movement<br>**Institutional power structures**<br>- e.g., criminal justice system, education, marriage
	**Historical**	**Intergenerational power**<br>- at the macro, meso and micro levels<br>**Legacy of institutional power structures**<br>- e.g., Colonialism, political institutions legitimization of slavery<br>**Legacy of discriminatory laws/policies/practices**<br>- e.g., greater alcohol outlet density in historically redlined communities(11)<br>- Changes in the recognition of cisgender identities<br>**Legacy of grassroots movements**<br>- e.g., the Temperance movement (~1820-1930s), which was largely driven by women protesting against the harm they suffered due to men's drinking (12,13)

<table>
<tr><th></th><th></th><th>DOMAIN OF INFLUENCE</th></tr>
<tr><th></th><th></th><th>BEHAVIOUR</th></tr>
<tr><td rowspan="4">LEVEL OF INFLUENCE</td><td>Individual</td><td>Alcohol use & motivations
- Quantity and frequency of consumption
- Pattern of drinking and drinking settings e.g. regular versus heavy episodic drinking, solitary versus ‘social’ drinking (14), drinking at home versus in a bar
- Psychological predictors of alcohol use e.g. alcohol expectancies (15) and motivations for drinking (16), perceptions of own drinking behaviour
- Beverage preferences (17)
- Affordability (in relation to individual SES)
- Age of drinking onset (18)
Psychology
- Other psychological variables influence on alcohol consumption, such as, world assumptions, pre-meditation, impulsivity etc. (19)
- Variation over the life course e.g., changes in sensation seeking with age
Coping strategies
- Those exposed to increased stressors/discrimination/stereotype-threat may be pushed off a ‘healthy’ trajectory via harmful internalising (e.g., anxiety, depression, drinking to cope, self-harm) and/or externalising (e.g., aggression) responses (20)
- Individuals may develop ‘healthy’/ ‘constructive’ responses e.g., active resistance/ engagement in advocacy, help-seeking behaviour as well as or instead of the above
Other behaviours
- Interaction between alcohol use and other health behaviours, e.g., smoking, diet (21), sleep hygiene (22);
- Other hobbies/leisure activities which may influence desires to drink (23);
- Behaviours whilst intoxicated e.g., driving.</td></tr>
<tr><td>Interpersonal</td><td>Interpersonal coping
- For example, the appraisal of stressors as "our" problem, rather than "mine" or "yours," is associated with less psychological distress in couples (24,25).
Family functioning
- Family/ parent-caregiver relations can influence alcohol consumption in adolescence and early adulthood (26)
- Family cohesion
- Childcare responsibilities
- Interpersonal violence/ domestic abuse
- Family rejection
- Intergenerational social mobility
- Incarcerated relative(s)
School/Work functioning
- Employment contract & working hours (length, regularity, sociable vs unsociable, flexibility, type and intensity of work, autonomy) – may influence stress, time to attend health appointments, etc.
- Direct or vicarious exposure to workplace interpersonal discrimination/ sexual harassment
- Work-based support (e.g., pastoral care, sick leave, employment-based health insurance)</td></tr>
<tr><td>Community</td><td>Local laws and policies
- State level alcohol policies e.g., regarding drink prices/taxes/distribution ('wet' versus 'dry' states),
- State level variation in other relevant laws and policies, e.g., Affordable Care Act; whether LGBTQ+ populations included in non-discrimination laws; gun laws etc.
- Local variation in actual enforcement of laws/ policies
Communal coping
- Communal or collectivistic cultures (e.g., any African, Asian, and Latin American) more likely to use collective coping methods, e.g., utilizing social support to deal with macro-level social stressors or, forbearance (minimizing/concealing problems to reduce burden on others) (see for example,(27–29)
Community functioning
- Community cohesion and social capital
- Collective behaviour e.g., prevalence of events in which alcohol is promoted, community response to people with alcohol use disorders, levels of crime
- Intracultural accusations of assimilation (e.g., shown to impact alcohol use and mental health amongst Hispanic men) (30,31)</td></tr>
<tr><td>Societal</td><td>National laws and policies
- Alcohol related policies e.g. national minimal drinking age; health warning labelling; drink driving limits
- Other relevant laws and policies e.g. non-discrimination laws; Affordable Care Act</td></tr>
</table>

	Historical	**Historical trauma/ intergenerational transmission of trauma**<br>- May influence alcohol consumption directly or indirectly and in either direction (see, for example Gameon & Skewes, 2021; Pokhrel & Herzog, 2014)

		DOMAIN OF INFLUENCE
		**DIGITAL ENVIRONMENT**
**LEVEL OF INFLUENCE**	**Individual**	**Technology access, attitudes towards use, digital literacy, and self-efficacy**<br>- All can influence use of digital technologies<br>- Variation in digital health access and use across demographic groups (34,35)<br>- Use of digital technologies (such as online health information seeking, social media use for social support and app-based interventions), are broadly associated with positive health outcomes including adopting healthy behaviours (34)<br>- Variation in engagement across demographic groups (34,35)
	**Interpersonal**	**Implicit tech bias**<br>- E.g., evidence of differential provision of access to electronic patient portals (which provide individuals with access to their electronic health record and the ability to message their healthcare providers).(36)<br>**Online discrimination**<br>- Online racism, both direct and vicarious, linked to psychological distress, alcohol use severity, and loneliness. (37,38)<br>**Interdependence**<br>- Reliance on others for digital skills/ access/ equipment<br>- Intergenerational digital skill acquisition<br>**Patient-tech-clinician relationship**<br>- Digitization of healthcare may 'democratize' the relationship between patients and clinicians(39)
	**Community**	**Community infrastructure**<br>- Online alcohol sales and home delivery (geographical variation)(40)<br>**Digital health infrastructure**<br>- Availability and cultural appropriateness of digital alcohol interventions and screening(41,42)<br>- Digital redlining – the systematic process procedure through which certain groups are denied equitable access to digital tools like the internet – leading to inequities in educational/ employment opportunities, and healthcare services /information healthcare. (43,44)<br>**Community tech norms**<br>- Level of congruence between the tech norms of a particular community and that of the dominant culture/ that used for e-health interventions<br>**Community partners**<br>- For example, tech advocacy groups, digital literacy training programs.
	**ADDIN ZOTERO_ITEM CSL_CITATION**	**Algorithmic bias**<br>- For example, use of race correction in clinical algorithms may delay referral to specialist care, receipt of treatment etc.(45)<br>**Tech policy and design standards**<br>- For example, presence and enforcement of standards/guidelines to make web content more accessible.<br>**Digital Alcohol Marketing**<br>- Exposure to alcohol marketing through digital media (e.g. social networking media and downloadable apps) appears associated with higher levels of drinking behaviour (Lobstein et al., 2017)<br>- Engagement with digital alcohol marketing also associated with increased alcohol consumption, binge drinking and hazardous drinking (Noel et al., 2020)
	**Historical**	**Digital health as a paradigm shift**<br>- Increasing integration of technological advancements in healthcare and health systems, bringing both benefits (e.g., reversing an old paradigm of paternalistic model of medicine) and risks (e.g., potential to exclude specific groups, unregulated/disruptive technologies) (39,48)

		DOMAIN OF INFLUENCE
		**PHYSICAL/ BUILT ENVIRONMENT**
LEVEL OF INFLUENCE	**Individual**	**Personal environment**<br>- Home occupancy status/ housing stability/quality (49)<br>- Sleep environment<br>- Prison conditions (incarcerated population)<br>- Weather/ availability of shelter (homeless population)
	**Interpersonal**	**Household environment**<br>- Size of household (e.g., overcrowding, social isolation); internet access (for interpersonal communication); incarceration<br>**School/Work Environment**<br>- E.g., working from home versus vs. office vs. manual/skilled labour, public vs boarding school: influencing availability of alcohol and repercussions of drinking
	**Community**	**Community environment & resources**<br>- *Alcohol specific:*<br>Neighbourhood variation in alcohol environment, including differences in: alcohol outlet density & outlet types (50); alcohol advertising (51); alcohol product ranges and prices (e.g. low cost, high-strength malt liquor readily available and widely promoted in poor, inner-city neighbourhoods, especially African-American neighbourhoods (52,53)); availability of illicit alcohol; home delivery of alcohol; the location of publicly funded alcohol treatment programmes (54).<br>Government investment in localities (depending on the level of income in the locality)<br>- *Other:*<br>Neighbourhood variation in availability and quality of educational institutions, housing, public transport, green-space, safe and accessible public facilities and (non-alcohol-centric) leisure activities. Neighbourhood variation in exposure to law enforcement, guns, psychoactive substances, environmental pollutants, and other hazards e.g., open water, unsafe sites.<br>**Environments that facilitate gendered drinking:**<br>- Certain social venues actively promote alcohol use as a performance of hegemonic masculinity. For example, male-centric venues such as sports bars, student bars and fraternity houses often normalize heavy drinking among men (21,22). However, research on college students suggest that this might be most relevant for White students, as some minoritized racial and ethnic groups are found to be less swayed by heavy drinking norms on campus (55).<br>**Residential segregation**<br>- Racial residential segregation considered a fundamental cause of health disparities (56). Level of segregation associated with inequalities in poverty, income, home value, homeownership and with political polarization.(56,57).
	**Societal**	**Urban design**<br>- Accessibility and inclusivity; distribution of commercial/industrial/recreational areas<br>**Public housing provision**<br>**Criminal justice system**<br>- Differential treatment within the criminal justice system based on race and ethnicity, resulting in inequities in incarceration rates; Influence of Prison Industrial Complex (58)<br>**Transport inequity**<br>- Influencing, for example, access to work (59), safety at night/ when intoxicated (60)<br>**Organisation of the government**<br>- E.g., Gerrymandering (political manipulation of electoral district boundaries), often at the expense of ethnic minority communities, reducing their influence on policy (alcohol related or otherwise).
	**Historical**	**Legacy of discriminatory design**<br>- Social environment (e.g., safety, public transport) designed around requirements of majority groups (particularly White men)<br>- Present day risks for exposure to retail alcohol availability delimited by historic exclusionary land use practices (61).<br>- Public parks created for ‘moral improvement', not for 'immoral' activities such as drinking.<br>**Legacy of pandemics and related policies**<br>- E.g. COVID-19 resulting in increased working from home, increased availability of home delivery alcohol and heightened inequities in the education system (62)<br>- E.g. HIV associated with alcohol use disorders and AUD can influence the spread of HIV (63,64)

		DOMAIN OF INFLUENCE
		**SOCIOCULTURAL ENVIRONMENT**
**LEVEL OF INFLUENCE**	**Individual**	**Language**<br>- Influencing ability to engage with public services, development of social networks etc.<br>**Identity**<br>- High racial/ethnic/cultural identity may increase the association between discrimination and substance use and/or act as a buffer. This appears to vary by the strength of identity and specific racial/ethnic group (Woo et al., 2019)<br>**Religion**<br>- Associated drinking norms, including variation by religious subtypes e.g., Catholic vs Protestant<br>**Assimilation/ acculturation**<br>- Acculturative stress<br>- Adoption of the dominant culture's drinking norms<br>- Segmented assimilation influencing social mobility/outcomes
	**Interpersonal**	**Interpersonal discrimination**<br>- Exposure to non-verbal or verbal discrimination/ microaggressions/ hate crimes<br>- Impact of childhood/adolescent bullying (for both victims and bullies) (66)<br>**Family/ workplace norms**<br>- Norms re. drinking / non-alcohol centric ways of socialising<br>- Norms re. seeking help (for drinking related problems)<br>- Family role expectations/ level of caring responsibilities<br>- Drinking norms and opportunities related to type of work (e.g., office versus manual jobs) or specific setting (e.g., at College)<br>- Historical family experiences with alcohol/alcoholism<br>- Social acceptability of drinking at/after work<br>**Social network**<br>- Peer group composition/ social network diversity<br>- Availability of mentors/role models/beneficial social connections, influencing ability to cope with stressors/ resolve problems (fundamental cause theory)<br>- Religious/cultural norms / pressures (e.g., to not drink at all)
	**Community**	**Local structural discrimination**<br>- Differences in average income by geographical areas (influencing relative affordability of alcohol)<br>- Discrimination in the local criminal justice system: many laws disproportionately affecting Black people/ people with low SES (e.g., civil citations for wearing 'saggy pants'); over policing and racial profiling by police (e.g., Black people more likely to be stopped/ charged for crimes alcohol-related or otherwise); differential impact of holding a criminal record (on for example, housing, employment) based on intersection with other characteristics (e.g., race and ethnicity); positive association between exposure to policing and substance use amongst Black youth.<br>- Variation in local implementation of policy/regulations (e.g., enforcement legal drinking age)<br>- Discriminatory housing and mortgage practices<br>- Discriminatory hiring/firing practices<br>- Varying degrees of social support/ 'safety net'<br>**Community norms**<br>- Norms re. drinking / non-alcohol centric ways of socialising (e.g., religious norms)<br>- Norms re. seeking help for (drinking) related problems (gendered)<br>- level of exposure to drinking opportunities within community

	Societal	**Social norms**<br>- Strong gendered injunctive norms on alcohol use, e.g., men and students expected to drink heavily, women expected to moderate their consumption (varying at intersections of ethnicity/SES/age);<br>- classification of drinking behaviour based on societal stereotypes (e.g., same drinking considered AUD in certain individuals and not others)<br>- stigmatisation of individuals perceived to have AUDs<br>- location of drinking influences whether it is considered immoral/penalised (e.g., drinking from a bottle in a park versus drinking a glass of wine in a restaurant);<br>- differential perception of 'drunkenness' conditional on race and ethnicity<br>- associations of certain drinking practices with (aspirational) class<br>- differences in beverage preferences conditional on class expectations;<br>- differences in cultural norms around children drinking (which may subsequently influence their drinking in adulthood)<br>- Public stigmatization of persons with alcohol use disorders (3)<br>- Differences between collectivist and individualistic cultures (67)<br>**Social role expectations**<br>- e.g., gender roles, expectations of employment / relationships/ parenting (varying by age/ gender). Level of (in)congruence with social role expectations (and subsequent coping)<br>**Institutional and structural discrimination**<br>- State-level income inequality (Black–White and Hispanic–White poverty ratios) associated with alcohol problems (68)<br>- Persistent pay gap (by gender, race, and ethnicity)<br>- Targeted alcohol advertising to promote consumption in some groups (e.g. women, Black people, youth) (69)<br>- Different allowances for how spaces can/should be used based on age (e.g., use of parks);<br>- Discriminatory banking and lending practices<br>- Inadequate enforcement of existing anti-discrimination laws<br>- Ineffective collection, use and dissemination of disaggregated data<br>**Commercial determinants / alcohol industry**<br>- E.g., Alcohol industry encouraging emphasis on individual responsibility over regulatory policy(70)
	Historical	**Period-specific cultural patterns/ ‘zeitgeists’**<br>- A current sense of ‘collective discontent with society’ (71)<br>- Increased perceived polarization associated with incident anxiety and depression (72)<br>**Legacy of historical laws/policies/practices/beliefs**<br>- Alcohol prohibition (1920-1933) - leading to a rise in organized crime<br>Forced disconnection from traditional practices<br>Jim Crow laws e.g., redlining, mortgage discrimination (~1870-1960s)<br>Creation of criminal justice system inherently ‘unfair’<br>Women not allowed to vote prior to 1920<br>- September 11th terrorist attacks (911) leading to islamophobia, associated with mental health problems for Muslims in the US<br>Same-sex marriages not legalised in all states until 2015<br>- Historical stereotypes, persistent despite recent changes (e.g., perception of heavy drinking amongst young people at odds with recent decline in youth drinking).<br>- Minimal changes in public stigmatization of alcohol dependence over time (73)<br>Polygeny as a prevailing theory of racial origin<br>Historically heavy drinking in office jobs (white, male dominance)<br>- Increasing digitization of society<br>- Racial and ethnic discrimination against Asian Americans during the COVID-19 pandemic (74,75)

		DOMAIN OF INFLUENCE
		**HEALTHCARE SYSTEM**
LEVEL OF INFLUENCE	**Individual**	**Personal resources**<br>- Influencing access to healthcare e.g., money, time, insurance coverage<br>**Health literacy**<br>- Influenced by first language, literacy, level of education etc.<br>**Healthcare preferences**<br>- E.g., belief in, acceptability of, and engagement with traditional services (including alcohol screening and brief interventions)<br>- Use of ‘alternative’ (non-biomedical) therapies
	**Interpersonal**	**Discriminatory medical care**<br>- Differential likelihood of screening/intervention for AUDs (76).<br>- Differential use of stigmatising diagnoses (e.g., use of 'alcoholic liver cirrhosis' versus 'liver cirrhosis') and/or stigmatising language in relation to addictions (77), potentially as a result of other prejudices (e.g., racism/sexism/classism)<br>- Differential pain management, influenced by client and provider race, ethnicity, and gender (78–80)<br>- Care dependent upon individual’s quality/type of health insurance<br>**Provider-client interactions**<br>Influenced by:<br>- Provider/client conscious or subconscious discrimination and biases (e.g., women expected to drink less, stigmatisation of alcohol use disorders<br>- Time pressures, particularly in the presence of multiple complex health needs (ageism/ableism)<br>- Level of provider knowledge about drinking and comfort level in discussing drinking<br>- Power relations between patient and provider<br>- Provider's cultural competence<br>- Patient autonomy during interactions (e.g., influence of family members)
	**Community**	**Availability and quality of local healthcare (alcohol-related or otherwise)**<br>- Influenced by state level variability in implementation of healthcare laws/policies, e.g. Affordable Care Act<br>- Availability of services, including alcohol screening & brief interventions, and treatment services<br>- Quality of care<br>- Accessibility of healthcare facilities (including for people with disabilities)<br>- Public transport routes to/from health facilities<br>- Availability of culturally relevant support<br>- Availability of non-biomedical healthcare
	**Societal**	**Healthcare norms**<br>- Biases within medical textbooks e.g. gender bias (81), absence of skin tone diversity in medical textbook imagery (82)<br>- Biases within scientific peer review processes (83)<br>- Focus on HIV for some groups (e.g., LGBTQ+) may mask other medical needs<br>- Lack of understanding of healthcare issues outside of the 'normative' (e.g., heteronormative) realm<br>**Healthcare policies and practices**<br>- No system of universal healthcare in US<br>- Reducing budget for AUD<br>- Differences in availability and cost of health insurance conditional on age (84)<br>**Availability, quality, and affordability of health insurance**
	**Historical**	**Legacy of discriminatory biomedical healthcare beliefs and practices**<br>- Belief that Black people (particularly Black women) experience less pain(85)<br>- Medical Experimentation on Black Americans e.g., experimental gynaecological surgery exclusively on black slaves(85) (Washington 2008)<br>- Western-centric, biomedical model of health<br>- White, heteronormative, men historically the 'default person' in medicine

		DOMAIN OF INFLUENCE
		**BIOLOGY**
**LEVEL OF INFLUENCE**	**Individual**	**Biological mechanisms**<br>- Allostatic load/ biological embedding/ 'weathering' i.e., cumulative burden of chronic stress and major life events (e.g., sexism, racism, economic deprivation)<br>- Life course accumulation of physical trauma/comorbidities/chronic pain<br>- Neurobiological variation (e.g. genetic polymorphisms, endophenotypes) in sensitivity to contextual signal and cues (86)<br>- Maladaptive stress responses<br>- Telomere length (short telomere length associated with increased progression of chronic liver disease)<br>**Biological vulnerability**<br>- Genetics e.g., alcohol metabolism (ALDH2 deficiency common amongst East Asian people), genetic predisposition to mental health conditions; differential susceptibility to the environment/ biological sensitivity to context (for better and for worse) (86)<br>- Other comorbidities and their interaction with alcohol (e.g. diabetes, susceptibility to pathogens (87))<br>- Microbiome/ early-life gut microbiota (e.g., imbalances associated with adult disease outcomes including anxiety and depression)<br>- Disability status, including Foetal Alcohol Spectrum Disorders
	**Interpersonal**	**Caregiver-child interactions**<br>- Can influence cognitive, emotional, and neurobiological development and health outcomes in later life (88)<br>**Family microbiome**<br>- The gut microbiome influenced by individuals who share a household, with the microbiome increasingly recognized as having a fundamental role in physiology and health (89,90), and potentially with the progression of alcohol-related liver disease<br>**In-utero alcohol exposure**<br>- Associated with a range of negative health outcomes for the child, such as Fetal Alcohol Spectrum Disorder (91–93)<br>**Adverse childhood events**<br>- Experiencing two or more adverse childhood events significantly increases the risk for lifetime alcohol dependence (94)
	**Community**	**Community illness exposure**<br>- Prevalence of, and exposure to, communicable diseases (e.g., COVID-19, HIV) and interactions with alcohol use<br>**Herd immunity**
	**Societal**	**Immunizations/ vaccine uptake**<br>- Vaccine uptake considerably lower amongst people with substance use disorder (95)<br>**Pathogen exposure**<br>- Chronic and acute alcohol use can increase individual susceptibility to infections caused by bacterial and viral pathogens (87)
	**Historical**	**Intergenerational stress pathways**<br>- Chronic stress and alcohol exposure have cross-generational alternations in stress, anxiety, and depression-related behaviours, and potentially affect other health-related outcomes such as cancer risk (96)<br>**Influence of migration on the genetic structure of the US population**<br>- Intra- and inter- national migration influencing population level genetics (97)<br>- e.g., distribution of genes related to alcohol metabolism, alcohol use disorders and related traits (98)

References

1. Chartier K, Vaeth P, Caetano R. Focus On: Ethnicity and the Social and Health Harms From Drinking. Alcohol Res. 2014;35(2):229–37.
2. Gilbert PA, Zemore SE. Discrimination and Drinking: A Systematic Review of the Evidence. Soc Sci Med. 2016 Jul;161:178–94.
3. Kilian C, Manthey J, Carr S, Hanschmidt F, Rehm J, Speerforck S, et al. Stigmatization of people with alcohol use disorders: An updated systematic review of population studies. Alcohol Clin Exp Res. 2021 May;45(5):899–911.
4. Castro FG, Stein JA, Bentler PM. Ethnic Pride, Traditional Family Values, and Acculturation in Early Cigarette and Alcohol Use Among Latino Adolescents. J Prim Prev. 2009 Jul;30(3–4):265.
5. Opara I, Lardier DT, Boyd D, Boateng ACO, Brawner BM. The Association Between Racial Attitudes, Alcohol Use and Mood Disorders Among Black Adolescents. J Prev (2022). 2023 Feb;44(1):85–104.
6. Shim JK. Cultural Health Capital: A Theoretical Approach to Understanding Health Care Interactions and the Dynamics of Unequal Treatment. J Health Soc Behav. 2010 Mar 1;51(1):1–15.
7. Gilens M. Affluence and Influence: Economic Inequality and Political Power in America [Internet]. Princeton University Press; 2012 [cited 2025 Jan 2]. Available from: https://www.degruyter.com/document/doi/10.1515/9781400844821/html
8. Institute of State and Regional Affairs, Penn State Harrisburg. Pennsylvania's Stigma Reduction Opioid Behavior Change Campaign, Data Briefs: Community Based Organizations. Sponsored by the Pennsylvania Department of Drug and Alcohol Programs. [Internet]. 2023 [cited 2023 Nov 26]. Available from: https://storymaps.arcgis.com/stories/25936dcb2ec64636aec55c32e003618c
9. Garcia V, Lambert E, Fox K, Heckert D, Pinchi NH. Grassroots interventions for alcohol use disorders in the Mexican immigrant community: A narrative literature review. J Ethn Subst Abuse. 2022;21(3):773–92.
10. Herd D. Community Mobilization and the Framing of Alcohol-Related Problems. Int J Environ Res Public Health. 2010 Mar;7(3):1226–47.
11. Haley S, Jardine S, Kelvin E, Herrmann C, Maroko A. Neighborhood Alcohol Outlet Density, Historical Redlining, and Violent Crime in NYC 2014–2018. International Journal of Environmental Research and Public Health. 2023 Feb 12;20:3212.
12. Levine HG. Temperance and Women in 19th-Century United States. In: Kalant OJ, editor. Alcohol and Drug Problems in Women [Internet]. Boston, MA: Springer US; 1980 [cited 2025 Apr 19]. p. 25–67. Available from: https://doi.org/10.1007/978-1-4615-7737-9_2
13. Salway S. America's black temperance movement, 1827–1894: charting a forgotten history. American Nineteenth Century History. 2023 May 4;24(2):119–44.
14. Skrzynski CJ, Creswell KG. A systematic review and meta-analysis on the association between solitary drinking and alcohol problems in adults. Addiction. 2021;116(9):2289–303.

15. Treloar H, Piasecki TM, McCarthy DM, Sher KJ, Heath AC. Ecological evidence that affect and perceptions of drink effects depend on alcohol expectancies. Addiction. 2015;110(9):1432–42.
16. Cooke R, Crawford J. Psychological Predictors of Alcohol Consumption. In: Cooke R, Conroy D, Davies EL, Hagger MS, de Visser RO, editors. The Palgrave Handbook of Psychological Perspectives on Alcohol Consumption [Internet]. Cham: Springer International Publishing; 2021 [cited 2023 Nov 26]. p. 77–104. Available from: https://doi.org/10.1007/978-3-030-66941-6_4
17. Michael S, Naimi T, Matthews J, Nelson D. Alcoholic Beverage Preferences and Associated Drinking Patterns and Risk Behaviors Among High School Youth. American journal of preventive medicine. 2011 Apr 1;40:419–26.
18. Hingson RW, Heeren T, Winter MR. Age at Drinking Onset and Alcohol Dependence: Age at Onset, Duration, and Severity. Archives of Pediatrics & Adolescent Medicine. 2006 Jul 1;160(7):739–46.
19. Latzman RD, Chan WY, Shishido Y. Impulsivity moderates the association between racial discrimination and alcohol problems. Addict Behav. 2013 Dec;38(12):2898–904.
20. Gerrard M, Gibbons FX, Fleischli ME, Cutrona CE, Stock ML. Moderation of the effects of discrimination-induced affective responses on health outcomes. Psychology & Health. 2018 Feb 1;33(2):193–212.
21. Burton R, Fryers PT, Sharpe C, Clarke Z, Henn C, Hydes T, et al. The independent and joint risks of alcohol consumption, smoking, and excess weight on morbidity and mortality: a systematic review and meta-analysis exploring synergistic associations. Public Health. 2024 Jan 1;226:39–52.
22. Roehrs T, Roth T. Insomnia as a path to alcoholism: tolerance development and dose escalation. Sleep. 2018 May 12;41(8):zsy091.
23. Finlay AK, Ram N, Maggs JL, Caldwell LL. Leisure Activities, the Social Weekend, and Alcohol Use: Evidence From a Daily Study of First-Year College Students. J Stud Alcohol Drugs. 2012 Mar;73(2):250–9.
24. Rohrbaugh MJ, Shoham V, Skoyen JA, Jensen M, Mehl MR. We-talk, communal coping, and cessation success in a couple-focused intervention for health-compromised smokers. Fam Process. 2012 Mar;51(1):107–21.
25. Rentscher KE, Soriano EC, Rohrbaugh MJ, Shoham V, Mehl MR. Partner Pronoun Use, Communal Coping, and Abstinence during Couple-Focused Intervention for Problematic Alcohol Use. Fam Process. 2017 Jun;56(2):348–63.
26. Gutman LM, Eccles JS, Peck S, Malanchuk O. The influence of family relations on trajectories of cigarette and alcohol use from early to late adolescence. Journal of Adolescence. 2011 Feb 1;34(1):119–28.
27. Richman JA, Brown RL, Rospenda KM. The Great Recession and Drinking Outcomes: Protective Effects of Politically Oriented Coping. J Addict. 2014;2014:646451.
28. Gillig T, Macary J, Price R. Virtual Camp: LGBTQ Youths' Collective Coping During the COVID-19 Pandemic. International Journal of Communication. 2022 Jan 30;16:981–1005.

29. Moore III JL, Constantine MG. Development and initial validation of the Collectivistic Coping Styles Measure with African, Asian, and Latin American international students. Journal of Mental Health Counseling. 2005;27(4):329–47.
30. Thornhill CW, Castillo LG, Piña-Watson B, Manzo G, Cano MÁ. Mental health among Latinx emerging adults: Examining the role of familial accusations of assimilation and ethnic identity. J Clin Psychol. 2022 May;78(5):892–912.
31. Cano MÁ. Intracultural accusations of assimilation and alcohol use severity among Hispanic emerging adults: Moderating effects of acculturation, enculturation, and gender. Psychol Addict Behav. 2016 Dec;30(8):850–6.
32. Pokhrel P, Herzog TA. Historical Trauma and Substance Use among Native Hawaiian College Students. Am J Health Behav. 2014 May;38(3):420–9.
33. Gameon JA, Skewes MC. Historical trauma and substance use among American Indian people with current substance use problems. Psychology of Addictive Behaviors. 2021;35(3):295–309.
34. Hegeman PC, Vader DT, Kamke K, El-Toukhy S. Patterns of digital health access and use among US adults: a latent class analysis. BMC Digital Health. 2024 Jul 25;2(1):42.
35. Kontos E, Blake KD, Chou WYS, Prestin A. Predictors of eHealth Usage: Insights on The Digital Divide From the Health Information National Trends Survey 2012. J Med Internet Res. 2014 Jul 16;16(7):e172.
36. Ancker JS, Barrón Y, Rockoff ML, Hauser D, Pichardo M, Szerencsy A, et al. Use of an Electronic Patient Portal Among Disadvantaged Populations. J GEN INTERN MED. 2011 Oct 1;26(10):1117–23.
37. Keum BT, Ángel Cano M. Online racism, depressive and anxiety symptoms, coping-related drinking motives, and alcohol use severity among Black, Latina/o/x, and Asian emerging adults. Addictive Behaviors. 2023 Jan 1;136:107468.
38. Keum BT, Li X. Online Racism, Rumination, and Vigilance: Impact on Distress, Loneliness, and Alcohol Use. The Counseling Psychologist. 2023 Apr 1;51(3):422–48.
39. Meskó B, Drobni Z, Bényei É, Gergely B, Győrffy Z. Digital health is a cultural transformation of traditional healthcare. Mhealth. 2017 Sep 14;3:38.
40. Colbert S, Wilkinson C, Thornton L, Feng X, Richmond R. Online alcohol sales and home delivery: An international policy review and systematic literature review. Health Policy. 2021 Sep 1;125(9):1222–37.
41. McNeely J, McLeman B, Gardner T, Nesin N, Amarendran V, Farkas S, et al. Implementation of substance use screening in rural federally-qualified health center clinics identified high rates of unhealthy alcohol and cannabis use among adult primary care patients. Addiction Science & Clinical Practice. 2023 Sep 20;18(1):56.
42. Nittas V, Daniore P, Chavez SJ, Wray TB. Challenges in implementing cultural adaptations of digital health interventions. Commun Med. 2024 Jan 5;4(1):1–5.

43. Perzynski AT, Roach MJ, Shick S, Callahan B, Gunzler D, Cebul R, et al. Patient portals and broadband internet inequality. Journal of the American Medical Informatics Association. 2017 Sep 1;24(5):927–32.
44. McCall T, Asuzu K, Oladele CR, Leung TI, Wang KH. A Socio-Ecological Approach to Addressing Digital Redlining in the United States: A Call to Action for Health Equity. Front Digit Health. 2022 Jul 18;4:897250.
45. Vyas DA, Eisenstein LG, Jones DS. Hidden in Plain Sight — Reconsidering the Use of Race Correction in Clinical Algorithms. New England Journal of Medicine. 2020 Aug 27;383(9):874–82.
46. Noel JK, Sammartino CJ, Rosenthal SR. Exposure to Digital Alcohol Marketing and Alcohol Use: A Systematic Review. J Stud Alcohol Drugs Suppl. 2020 Mar;(Suppl 19):57–67.
47. Lobstein T, Landon J, Thornton N, Jernigan D. The commercial use of digital media to market alcohol products: a narrative review. Addiction. 2017 Jan;112 Suppl 1:21–7.
48. Meskó B. COVID-19's Impact on Digital Health Adoption: The Growing Gap Between a Technological and a Cultural Transformation. JMIR Hum Factors. 2022 Sep 19;9(3):e38926.
49. Choi NG, Marti CN, Choi BY. Job loss, financial strain, and housing problems as suicide precipitants: Associations with other life stressors. SSM - Population Health. 2022 Sep 1;19:101243.
50. Romley JA, Cohen DA, Ringel JS, Sturm R. Alcohol and Environmental Justice: The Density of Liquor Stores and Bars in Urban Neighborhoods in the United States [Internet]. Alcohol Research Documentation, Inc.; 2007 Jan [cited 2022 Sep 22]. Available from: https://www.rand.org/pubs/reprints/RP1323.html
51. Wilson RT, Till BD. Targeting of Outdoor Alcohol Advertising: A Study Across Ethnic and Income Groups. Journal of Current Issues & Research in Advertising. 2012 Aug 16;33(2):267–81.
52. Alaniz ML, Wilkes C. Pro-drinking messages and message environments for young adults: the case of alcohol industry advertising in African American, Latino, and Native American communities. J Public Health Policy. 1998;19(4):447–72.
53. Jones-Webb R, McKee P, Hannan P, Wall M, Pham L, Erickson D, et al. Alcohol and malt liquor availability and promotion and homicide in inner cities. Subst Use Misuse. 2008;43(2):159–77.
54. Jacobson JO, Robinson P, Bluthenthal RN. A multilevel decomposition approach to estimate the role of program location and neighborhood disadvantage in racial disparities in alcohol treatment completion. Soc Sci Med. 2007 Jan;64(2):462–76.
55. McCabe BE, Lee DL, Viray T. Does Ethnicity Moderate the Link between Drinking Norms and Binge Drinking in College Students? Int J Ment Health Addict. 2019 Jun;17(3):493–501.
56. Williams DR, Collins C. Racial Residential Segregation: A Fundamental Cause of Racial Disparities in Health. Public Health Rep [Internet]. 2001 [cited 2023 Jun 14]; Available from: https://www-ncbi-nlm-nih-gov.sheffield.idm.oclc.org/pmc/articles/PMC1497358/

57. Menendian S, Gambhir S, Gailes A. The Roots of Structural Racism Project: Twenty-First Century Racial Residential Segregation in the United States [Internet]. Othering and Belonging Institute; 2023 [cited 2023 Dec 7]. Available from: https://belonging.berkeley.edu/roots-structural-racism
58. Klein DE, Lima JM. The Prison Industrial Complex as a Commercial Determinant of Health. Am J Public Health. 2021 Oct;111(10):1750–2.
59. Maharjan S, Janatabadi F, Ermagun A. Spatial Inequity of Transit and Automobile Access Gap across America for Underserved Population. Transportation Research Record. 2023 Jun 21;03611981231171914.
60. Jackson CK, Owens EG. One for the road: Public transportation, alcohol consumption, and intoxicated driving. Journal of Public Economics. 2011 Feb 1;95(1):106–21.
61. Lee JP, Ponicki W, Mair C, Gruenewald P, Ghanem L. What explains the concentration of off-premise alcohol outlets in Black neighborhoods? SSM Popul Health. 2020 Dec;12:100669.
62. Gee KA, Asmundson V, Vang T. Educational impacts of the COVID-19 pandemic in the United States: Inequities by race, ethnicity, and socioeconomic status. Current Opinion in Psychology. 2023 Aug 1;52:101643.
63. Duko B, Ayalew M, Ayano G. The prevalence of alcohol use disorders among people living with HIV/AIDS: a systematic review and meta-analysis. Substance Abuse Treatment, Prevention, and Policy. 2019 Nov 14;14(1):52.
64. Nguyen CT, Nguyen HT, Vu TMT, Le Vu MN, Vu GT, Latkin CA, et al. Mapping Studies of Alcohol Use Among People Living with HIV/AIDS During 1990–2019 (GAPRESEARCH). AIDS Behav. 2023 Dec 1;27(12):3981–91.
65. Woo B, Fan W, Tran TV, Takeuchi DT. The role of racial/ethnic identity in the association between racial discrimination and psychiatric disorders: A buffer or exacerbator? SSM - Population Health. 2019 Apr 1;7:100378.
66. Armitage R. Bullying in children: impact on child health. BMJ Paediatr Open. 2021;5(1):e000939.
67. Inman RA, da Silva SMG, Bayoumi RR, Hanel PHP. Cultural Value Orientations and Alcohol Consumption in 74 Countries: A Societal-Level Analysis. Frontiers in Psychology [Internet]. 2017 [cited 2024 Jan 4];8. Available from: https://www.frontiersin.org/articles/10.3389/fpsyg.2017.01963
68. Karriker-Jaffe KJ, C. M. Roberts S, Bond J. Income Inequality, Alcohol Use, and Alcohol-Related Problems. Am J Public Health. 2013 Apr;103(4):649–56.
69. Rhoades E, Jernigan DH. Risky messages in alcohol advertising, 2003-2007: results from content analysis. J Adolesc Health. 2013 Jan;52(1):116–21.
70. Yoon S, Lam TH. The illusion of righteousness: corporate social responsibility practices of the alcohol industry. BMC Public Health. 2013 Dec;13(1):1–11.
71. van der Bles AM, Postmes T, Meijer RR. Understanding Collective Discontents: A Psychological Approach to Measuring Zeitgeist. PLoS One. 2015 Jun 26;10(6):e0130100.
72. Nayak SS, Fraser T, Panagopoulos C, Aldrich DP, Kim D. Is divisive politics making Americans sick? Associations of perceived partisan polarization with

physical and mental health outcomes among adults in the United States. Social Science & Medicine. 2021 Sep 1;284:113976.
73. Pescosolido BA, Manago B, Monahan J. Evolving Public Views On The Likelihood Of Violence From People With Mental Illness: Stigma And Its Consequences. Health Affairs. 2019 Oct;38(10):1735–43.
74. Oh H, Zhou S, Banawa R. The mental health consequences of discrimination against Asian American/Pacific Islanders. Cai L Li, Ruiz, Zhou, editor. Psychiatric Services. 2021;72(11):1359.
75. McGarity-Palmer R, Saw A, Tsoh JY, Yellow Horse AJ. Trends in Racial Discrimination Experiences for Asian Americans During the COVID-19 Pandemic. J Racial Ethn Health Disparities. 2023 Jan 5;1–16.
76. Pinedo M. Missed opportunities by health care providers to reduce racial/ethnic disparities in the use of alcohol treatment services. Drug Alcohol Depend. 2021 Sep 1;226:108851.
77. Carpenter JE, Catalanotti J, Notis M, Brokus C, Moran TP, Akselrod H, et al. Use of nonstigmatizing language is associated with improved outcomes in hospitalized people who inject drugs. J Hosp Med. 2023 Aug;18(8):670–6.
78. Weisse CS, Sorum PC, Sanders KN, Syat BL. Do gender and race affect decisions about pain management? J GEN INTERN MED. 2001 Apr 1;16(4):211–7.
79. Weisse CS, Sorum PC, Dominguez RE. The influence of gender and race on physicians' pain management decisions. J Pain. 2003 Nov;4(9):505–10.
80. Cintron A, Morrison RS. Pain and Ethnicity in the United States: A Systematic Review. Journal of Palliative Medicine. 2006 Dec;9(6):1454–73.
81. Dijkstra AF, Verdonk P, Lagro-Janssen ALM. Gender bias in medical textbooks: examples from coronary heart disease, depression, alcohol abuse and pharmacology. Med Educ. 2008 Oct;42(10):1021–8.
82. Louie P, Wilkes R. Representations of race and skin tone in medical textbook imagery. Soc Sci Med. 2018 Apr;202:38–42.
83. Schick MR, Tomko RL, Maralit AM, Afzal Z, Squeglia LM, Freda A, et al. Gender parity and homophily in the Drug and Alcohol Dependence editorial process. Drug Alcohol Depend. 2022 Jul 1;236:109493.
84. Tipirneni R, Solway E, Malani P, Luster J, Kullgren JT, Kirch M, et al. Health Insurance Affordability Concerns and Health Care Avoidance Among U.S. Adults Approaching Retirement. JAMA Netw Open. 2020 Feb 5;3(2):e1920647.
85. Washington H. Medical Apartheid. THE DARK HISTORY OF MEDICAL EXPERIMENTATION ON BLACK AMERICANS FROM COLONIAL TIMES TO THE PRESENT. Knopf Doubleday Publishing Group; 2008.
86. Ellis BJ, Boyce WT. Differential susceptibility to the environment: toward an understanding of sensitivity to developmental experiences and context. Dev Psychopathol. 2011 Feb;23(1):1–5.
87. Molina PE, Happel KI, Zhang P, Kolls JK, Nelson S. Focus On: Alcohol and the Immune System. Alcohol Res Health. 2010;33(1–2):97–108.

88. Luby JL, Rogers C, McLaughlin KA. Environmental Conditions to Promote Healthy Childhood Brain/Behavioral Development: Informing Early Preventive Interventions for Delivery in Routine Care. Biological Psychiatry Global Open Science. 2022 Jul 1;2(3):233–41.
89. Day AW, Kumamoto CA. Gut Microbiome Dysbiosis in Alcoholism: Consequences for Health and Recovery. Front Cell Infect Microbiol [Internet]. 2022 Mar 3 [cited 2025 Jan 7];12. Available from: https://www.frontiersin.org/journals/cellular-and-infection-microbiology/articles/10.3389/fcimb.2022.840164/full
90. Rothschild D, Weissbrod O, Barkan E, Kurilshikov A, Korem T, Zeevi D, et al. Environment dominates over host genetics in shaping human gut microbiota. Nature. 2018 Mar;555(7695):210–5.
91. Ungerer M, Knezovich J, Ramsay M. In Utero Alcohol Exposure, Epigenetic Changes, and Their Consequences. Alcohol Res. 2013;35(1):37–46.
92. Mattson SN, Bernes GA, Doyle LR. Fetal Alcohol Spectrum Disorders: A Review of the Neurobehavioral Deficits Associated With Prenatal Alcohol Exposure. Alcoholism: Clinical and Experimental Research. 2019;43(6):1046–62.
93. Subramoney S, Eastman E, Adnams C, Stein DJ, Donald KA. The Early Developmental Outcomes of Prenatal Alcohol Exposure: A Review. Frontiers in Neurology [Internet]. 2018 [cited 2023 Nov 27];9. Available from: https://www.frontiersin.org/articles/10.3389/fneur.2018.01108
94. Pilowsky DJ, Keyes KM, Hasin DS. Adverse Childhood Events and Lifetime Alcohol Dependence. Am J Public Health. 2009 Feb;99(2):258–63.
95. Suffel AM, Ojo-Aromokudu O, Carreira H, Mounier-Jack S, Osborn D, Warren-Gash C, et al. Exploring the impact of mental health conditions on vaccine uptake in high-income countries: a systematic review. BMC Psychiatry. 2023 Jan 7;23(1):15.
96. Rice RC, Gil DV, Baratta AM, Frawley RR, Hill SY, Farris SP, et al. Inter- and transgenerational heritability of preconception chronic stress or alcohol exposure: Translational outcomes in brain and behavior. Neurobiology of Stress. 2024 Mar 1;29:100603.
97. Dai CL, Vazifeh MM, Yeang CH, Tachet R, Wells RS, Vilar MG, et al. Population Histories of the United States Revealed through Fine-Scale Migration and Haplotype Analysis. The American Journal of Human Genetics. 2020 Mar 5;106(3):371–88.
98. Edenberg HJ, Foroud T. Genetics and alcoholism. Nat Rev Gastroenterol Hepatol. 2013 Aug;10(8):487–94.
