# Supplementary material for: Applying an intersectional lens to alcohol inequities: A conceptual framework
Source: Addiction. 2025 Jul 14;120(12):2585–98. doi: 10.1111/add.70130 (PMC12586755; doi:10.1111/add.70130)
Supplement: Supplementary file 2 — Appendix S2. Supporting Information. [file ADD-120-2585-s001.docx]

Appendix 2 - Characteristics of workshop participants

|  | **Research interests** | **Gender** | **Age cat.** | **Race and ethnicity** | **Level of education** | **Country of birth** | **Country of residence** | **First language** |
| --- | --- | --- | --- | --- | --- | --- | --- | --- |
| 1 | - Health inequities - Intersectionality - Ageing | Male | 40-44 | White British | PhD | England | England | English |
| 2 | - Alcohol use and policy - Health Communication - Health equity - Intersectionality - LGBTQ+ Health | Female | 30-44 | Black/African American | PhD, MPH | United States | United States | English |
| 3 | - Alcohol-related disparities - Health equity - Healthcare access | Female | 50-64 | Asian American | DrPH | Indonesia | United States | English |
| 4 | - Alcohol use - Health inequities - Public health policy | Female | 15-29 | White | PhD | Germany | Denmark | German |
| 5 | - Alcohol/substance use - Health inequities - Public health policy | Female | 30-44 | White  German | PhD | Germany | Canada | German |
| 6 | - Health inequities - Health & social care inequities - Intersectionality | Female | 30-44 | White Brazilian | PhD | Brazil | England | Portuguese |
| 7 | - Health Inequities | Female | 45-50 | Black | PhD | Jamaica | England | English |
| 8 | - Alcohol policy - Health inequities - Alcohol epidemiology | Male | 30-44 | White British | MSc | England | England | English |
| 9 | - Alcohol epidemiology - Alcohol policy | Male | 30-44 | White | PhD | England | England | English |
| 10 | - Health behaviours - Health inequities - Public health policy | Male | 45-54 | White British | PhD | United Kingdom | United Kingdom | English |
| 11 | - Alcohol use - Health behaviours - Public health modelling | Female | 30-44 | White | PhD | England | England | English |
| 12 | - Health inequities - Intersectionality - Alcohol/ substance use | Female | 30-44 | White British | MSc | England | England | English |
